# The magic of biaryl linkers: the electronic coupling through them defines the propensity for excited-state symmetry breaking in quadrupolar acceptor–donor–acceptor fluorophores†

**DOI:** 10.1039/d3sc03812b

**Published:** 2023-11-06

**Authors:** John A. Clark, Damian Kusy, Olena Vakuliuk, Maciej Krzeszewski, Krzysztof J. Kochanowski, Beata Koszarna, Omar O'Mari, Denis Jacquemin, Daniel T. Gryko, Valentine I. Vullev

**Affiliations:** a Department of Bioengineering, University of California Riverside, 900 University Ave. Riverside CA 92521 USA vullev@ucr.edu; b Institute of Organic Chemistry, Polish Academy of Sciences Kasprzaka 44–52 01-224 Warsaw Poland dtgryko@icho.edu.pl; c Nantes Université, CNRS CEISAM UMR 6230 F-44000 Nantes France Denis.Jacquemin@univ-nantes.fr; d Institut Universitaire de France (IUF) F-75005 Paris France; e Department of Chemistry, University of California Riverside CA 92521 USA; f Department of Biochemistry, University of California Riverside CA 92521 USA; g Materials Science and Engineering Program, University of California Riverside CA 92521 USA

## Abstract

Charge transfer (CT) is key for molecular photonics, governing the optical properties of chromophores comprising electron-rich and electron-deficient components. In photoexcited dyes with an acceptor–donor–acceptor or donor–acceptor–donor architecture, CT breaks their quadrupolar symmetry and yields dipolar structures manifesting pronounced solvatochromism. Herein, we explore the effects of electronic coupling through biaryl linkers on the excited-state symmetry breaking of such hybrid dyes composed of an electron-rich core, *i.e.*, 1,4-dihydropyrrolo[3,2-*b*]pyrrole (DHPP), and pyrene substituents that can act as electron acceptors. Experimental and theoretical studies reveal that strengthening the donor–acceptor electronic coupling decreases the CT rates and the propensity for symmetry breaking. We ascribe this unexpected result to effects of electronic coupling on the CT thermodynamics, which in its turn affects the CT kinetics. In cases of intermediate electronic coupling, the pyrene-DHPP conjugates produce fluorescence spectra, spreading over the whole visible range, that in addition to the broad CT emission, show bands from the radiative deactivation of the locally excited states of the donor and the acceptors. Because the radiative deactivation of the low-lying CT states is distinctly slow, fluorescence from upper locally excited states emerge leading to the observed anti-Kasha behaviour. As a result, these dyes exhibit white fluorescence. In addition to demonstrating the multifaceted nature of the effects of electronic coupling on CT dynamics, these chromophores can act as broad-band light sources with practical importance for imaging and photonics.

## Abstract 2

Intermediate donor–acceptor electronic coupling leads to a brilliant fluorescence behaviour.

## Graphical abstract

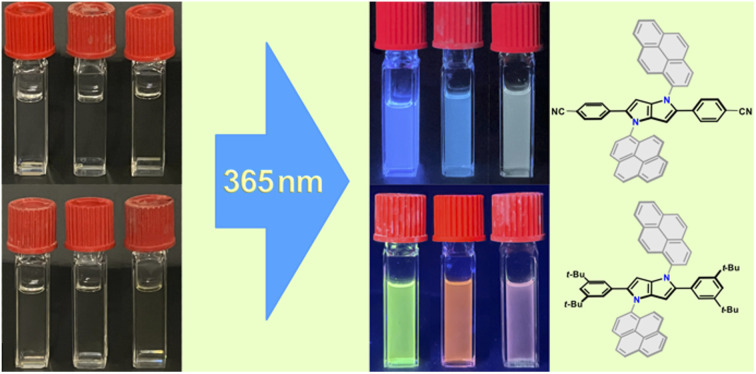

## Introduction

Charge transfer (CT) phenomena are omnipresent, governing energy conversion in both living and man-made systems, as well as the behaviour of molecular and nanometer-scale structures.^1^ The extent of charge separation (CS) in electronic excited states presenting a CT character affects the propensity of chromophores to undergo radiative deactivation and their susceptibility to medium polarity, which consequently defines their photoemission efficiencies and solvatochromic responses.^2–4^ Due to inherently large Stokes' shifts and solvatochromic properties, dyes with emissive CT states have proven indispensable for photonics, optical imaging, and biosensing.^5–10^ Solvatochromism offers a robust means for probing the polarity of microenvironments, where large emission quantum yields ensure the facility of such sensing.^11–13^ Centrosymmetric dyes with acceptor–donor–acceptor (A–D–A) and donor–acceptor–donor (D–A–D) architectures which form emissive CT states most commonly display solvatofluorochromic behaviour. That is, while an increase in solvent polarity induces bathochromic fluorescence shifts, it does not affect the absorption.^14–17^ This feature allows excitation at the same wavelength regardless the solvating media.

The thermodynamics of intramolecular charge transfer (ICT) depends on the energy levels of CT states and to what extent they are shifted upon altering solvent polarity.^18–20^ This focus on the ICT thermodynamics, due to its deterministic nature for solvatochromism, frequently leaves the donor–acceptor electronic coupling out of the discussion. Adiabatic ICT is a ubiquitous process that occurs between strongly coupled electron-rich and electron-deficient components of polarized, typically dipolar, photoexcited molecules.^21–24^ Weak electronic coupling between donors and acceptors, on the other hand, warrants a non-adiabatic (or diabatic) regime of CT.^1^

When optical-absorption electronic transitions of a bichromophoric dye are localized on its electron-rich component, *i.e.*, on the donor, the excited-state ICT involves electron transfer (ET) to the lowest unoccupied molecular orbital (LUMO) of the acceptor.^1,25–30^ Conversely, when the photoexcitation is localized on the electron-deficient component, *i.e.*, on the acceptor, of a bichromophoric dye, the ICT involves a hole transfer (HT) to the highest occupied molecular orbital (HOMO) of the donor.^1,25^ While a single-step HT is ET from the HOMO of a donor to a singly-occupied orbital of an acceptor, HT and ET are not the same. Mediated in the same electron donor–acceptor pair, HT and ET have different kinetics and different thermodynamics. The donor–acceptor electronic coupling along the HOMOs usually differs from that along the LUMOs. Also, excitation energies of the donor and the acceptor usually are not the same, rendering the driving forces for ET and HT different.^31^

Emerging from the paradigms of diabatic and adiabatic ICT, the regime of intermediate donor–acceptor electronic coupling requires special attention.^32–35^ The number of suitable structures for studying CT under such an intermediate-coupling regime is limited, which narrows down the possibilities for advancing the field.^36^ Searching for suitable structures in this regard, we focus on the biaryl linkers between aromatic substituents and 1,4-dihydropyrrolo[3,2-*b*]pyrroles (DHPPs).^37,38^

Among small aromatic heterocycles, DHPP is the most electron-rich, manifesting attractive optical and electronic properties. Centrosymmetric tetraarylpyrrolo[3,2-*b*]pyrroles (TAPPs) typically exhibit large fluorescence quantum yields and considerable Stokes' shifts. Such TAPPs, modified with electron-deficient substituents to form A–D–A structures, assume ground and Franck–Condon (FC) excited states with quadrupole symmetry.^39–41^ In the excited state, ICT between one of the acceptor units and the electron-rich core breaks the quadrupole symmetry and leads to dipolar structures, the emission wavelength of which is susceptible to changes of solvent polarity.^42^ That is, such A–D–A TAPPs exhibit solvatofluorochromism due to excited-state symmetry breaking (ES-SB).

The photophysical properties of TAPPs strongly depend not only on the nature, but also on the positions of their substituents.^41–43^ Numerous reports demonstrate that the electronic coupling through the carbons at positions 2 and 5 of the DHPP core can be particularly strong.^44,45^ Furthermore, computational studies reveal that dihedral angles between the core and aryl substituents at positions 2 and 5 decrease upon moving from the ground to the excited state, from *ca.* 50° to 30°, respectively.^46^ In the course of our studies, we have discovered that the electronic communication through the carbons at positions 3 and 6 is markedly weaker than that at positions 2 and 5.^40,47^

**Fig. 1:**
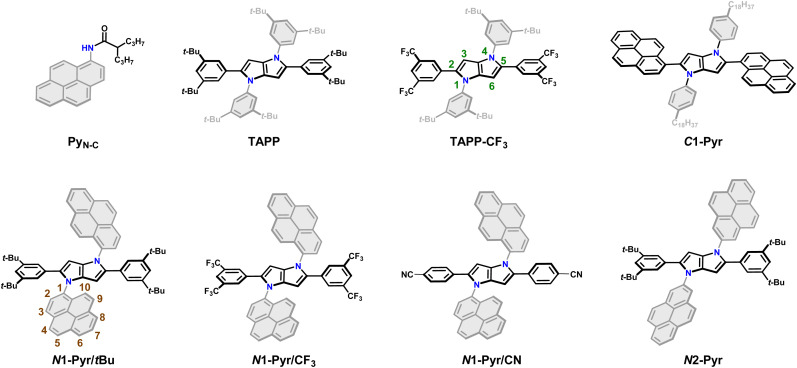
Chemical structures of the *C*-pyrenyl and *N*-pyrenyl substituted 1,4-dihydropyrrolo[3,2-*b*]pyrroles along with the model compounds.

The most intriguing sites for derivatization, however, prove to be the nitrogen atoms at positions 1 and 4 (Fig. 1). A common notion suggests there will be a decreased electronic coupling through pyrrole-type nitrogen atom which has left the effects of *N*-substituents of TAPPs largely unexplored. For instance, the dihedral angle of the *N*-phenyl substituents is typically less affected by the transition from the ground to the excited state.^46^ However, relevant to *N*-arylpyrroles, Rettig *et al.* reported that an increase in the strength of the electron donor lowers the energy of the CT state, resulting in broad CT-emission, even in hexane.^48^ In short, the nitrogen atoms of the pyrrolopyrrole core are promising but underexplored sites for the search of ICT under an intermediate electronic-coupling regime if proper aromatic electron acceptors attached to them.

Herein we select centrosymmetric TAPPs with A–D–A architectures possessing two pyrenyl units as electron acceptors. Regioisomerization of the biaryl links allows for variation of the donor–acceptor electronic coupling. Even in the weakly coupled A–D–A conjugates, the ICT is fast enough to ensure ES-SB. Nevertheless, enhancing the donor–acceptor electronic coupling in the pyrene-derivatized TAPPs starts preventing the ICT-driven ES-SB from occurring. As surprising as this result appears to be, it bears an important implication for the possible ways in which donor–acceptor coupling can affect the CT kinetics. In addition to a direct contribution to the electronic component of the rate constants, the electronic coupling can affect the energy levels of the CT state and hence the Franck–Condon (FC) nuclear contributions to the kinetics. The latter prevails in the case of the pyrene-substituted TAPPs with intermediately strong donor–acceptor electronic coupling. These conditions also allow formation of stable excited states that live long enough to produce not only CT emission, but also fluorescence from the locally excited pyrrolopyrrole donor and pyrene acceptors. Under such conditions, these conjugates produce white fluorescence with spectral bands spread over the whole visible region. Such behaviour of pyrene-substituted TAPPs is unprecedented for this class of compounds and demonstrates the emergence of synergy between donor–acceptor electronic coupling and CT thermodynamics. It shows new routes for exploring CT for energy science, organic electronics and photonics.

## Results

### Design rationale and synthesis

While *N*-arylpyrroles have been routinely prepared for decades, the recent emergence of DHPP and its TAPP derivatives presents entirely new photophysical opportunities. Specifically: (a) in contrast to pyrrole, DHPP is a distinct chromophore, exhibiting absorption and emission in the near ultraviolet (UV) and visible spectral regions; (b) TAPPs are centrosymmetric, making them prone to ES-SB.^41,49,50^

As evoked above, the electronic communication between the DHPP core of TAPPs and the aromatic substituents attached to the two nitrogen atoms is generally relatively weak. Phenyl derivatives at these positions hardly affect the resultant photophysics^42^ and any specific contribution from the benzene rings is hardly detectable. Therefore, we shift our attention to polycyclic aromatic hydrocarbons (PAHs) that may present energy levels of their frontier orbitals closer to those of the pyrrolopyrrole core. Such a resonance condition is a prerequisite for enhancing substituent effects. In this study we focus on DHPPs possessing pyrenes. This PAH is a well characterized chromophore,^51–58^ and many reports demonstrate its utility as an electron acceptor in CT systems of various complexity,^59–63^ which is complementary to the electron-rich DHPP core.

Employing our procedure we synthesized TAPPs with pyrenes attached to their nitrogen centres (*N*1-Pyr/*t*Bu and *N*2-Pyr, Fig. 1) from the corresponding aldehydes, amino-substituted PAHs and diacetyl, in yields of 10 to 40% (Scheme S1, see ESI† for details).^64^ The different carbon substituents, *i.e.*, di-*t*-butylphenyl, di-trifluoromethylphenyl and cyanophenyl (*N*1-Pyr/*t*Bu, *N*1-Pyr/CF_3_ and *N*1-Pyr/CN, Fig. 1), modulate the propensity of the pyrrolopyrrole core to act as an electron donor. Changing the position on the pyrene rings, at which they are attached to the nitrogen atoms, from 1 to 2, *i.e.*, *N*1-Pyr/*t*Bu*vs.N*2-Pyr (Fig. 1), further decreases the electronic coupling with the pyrrolopyrrole core because of the HOMO and LUMO nodes stretching from carbon 2 to carbon 7 of this PAH (Py_N-C_, Fig. 1).^65^

A TAPP with strongly coupled A–D–A structure, containing pyrenes attached to carbons 2 and 5 (*C*1-Pyr, Fig. 1), provides a control if the PAH induces ES-SB behaviour. In addition, TAPPs without PAH (TAPP and TAPP-CF_3_) and *N*-(pyren-1-yl)-2-propylpentanamide (Py_N-C_)^66^ provide a baseline for characterizing the properties of the electron-donor core and the electron-acceptor substituents. As electron-neutral substituents at positions 2 and 5 of the TAPPs, we selected 3,5-bis(*tert*-butyl)phenyls, *i.e.*, possessing rigid *t*-butyl solubilizing groups. Another choice involves 3,5-bis(trifluoromethyl)phenyl substituents, manifesting a slightly electron-deficient character. The importance of these design choices encompasses: (a) relatively “passive” phenyl moieties allowing the effects of the PAH *N*-aryl substituents to stand out; and (b) assurance of sufficient solubility in a broad range of solvents.

### Optical properties from steady-state spectroscopy

**Fig. 2:**
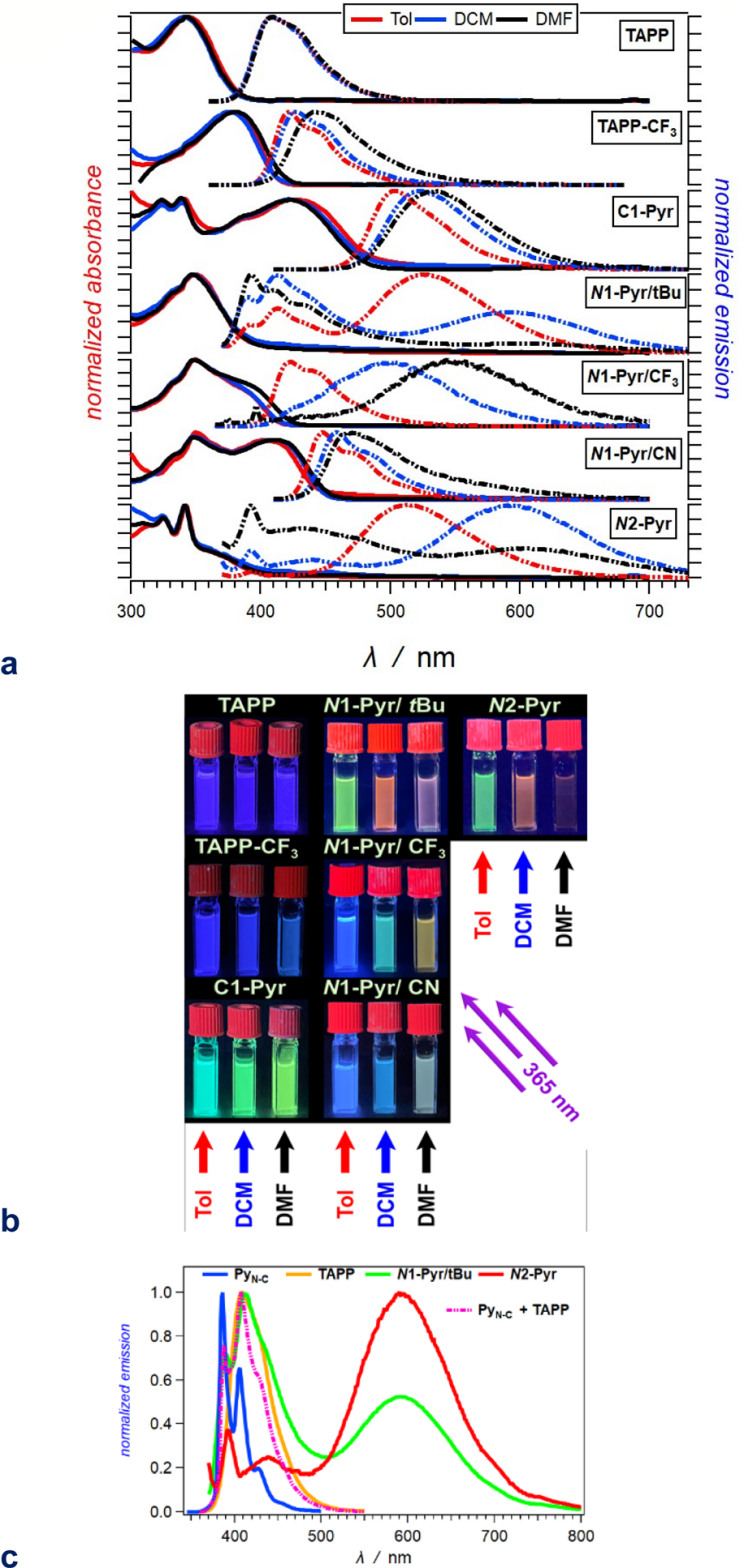
Optical properties of the TAPP derivatives. (a) Absorption (solid lines) and normalized emission (dashed lines) spectra of the compounds for toluene (red), DCM (blue) and DMF (black) (*λ*_ex_ = 350 nm for TAPP and TAPP-CF_3_; 360 nm for *N*1-Pyr/*t*Bu, *N*1-Pyr/CF_3_ and *N*2-Pyr; and 400 nm for *C*1-Pyr and *N*1-Pyr/CN). (b) Images of 20 μM solutions of these compounds in toluene (left columns), DCM (middle columns) and DMF (right columns), recorded under UV illumination (365 nm). (c) Superposition of the fluorescence spectra of *N*1-Pyr/*t*Bu and *N*2-Pyr with those of the TAPP and Py_N-C_ models carrying the pyrrolopyrrole and pyrene chromophores for DCM (Fig. 1).

Possessing neither electron-withdrawing substituents, nor π-expansion, TAPP serves as a primary model. It is a UV absorber exhibiting strong emission extending into the visible spectral region (Fig. 2a), *i.e.*, *λ*_abs_ ≈ 340 nm and *λ*_em_ ≈ 410 nm regardless of solvent polarity (Table 1), consistent with other simple tetraphenylpyrrolopyrroles.^64^

**Table 1:** Spectral properties of the dyes for solvents with different polarity

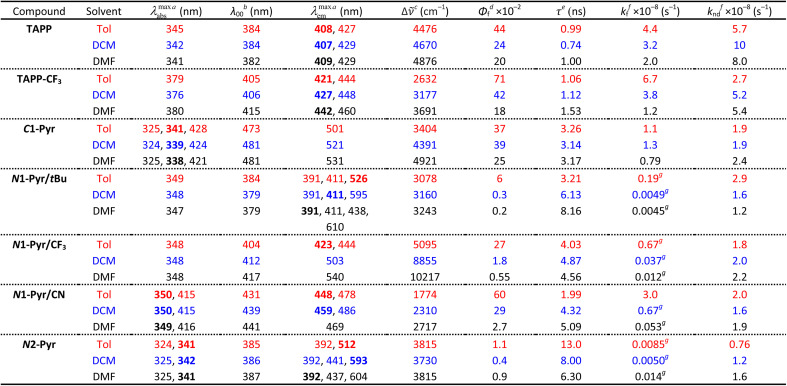

a Wavelengths of the maxima of the absorption and fluorescence spectra. The bold numbers correspond to the highest maxima for the spectra with multiple peaks (Fig. 2a).

b Wavelengths corresponding to the zero-to-zero energy, *ε*_00_, obtained from the crossing point between the absorption and the normalized fluorescence spectra, *i.e.*, *λ*_00_ = *h c ε*_00_^−1^.

c Stokes' shifts.

d Relative fluorescence quantum yields obtained by using ethanol solutions of coumarin 151 as standards.

e Excited-state lifetimes obtained from fluorescence decays measured at the spectral maxima.

f Rate constants of the radiative an non-radiative decays, *i.e.*, *k*_f_ = *Φ*_f_*τ*^−1^ and *k*_nd_ = (1 − *Φ*_f_)*τ*^−1^.

g Representing *k*_f_^(CT)^, *i.e.*, the radiative deactivation of the lowest lying CT states.

Replacing the 3,5-bis(*t*-Bu)C_6_H_3_ substituent at carbons 2 and 5 with the weakly electron-withdrawing 3,5-bis(CF_3_)C_6_H_3_ analogue alters the photophysical behaviour, as comparison between the spectral properties of TAPP and TAPP-CF_3_ reveals. The CF_3_ moieties induces a 35 nm bathochromic shift in the absorption, while only 15 to 30 nm in the emission – a trend reproduced by theory (*vide infra*). While the absorption of this A–D–A chromophore shows a negligible solvent dependence, an increase in media polarity induces bathochromic shifts of the fluorescence of TAPP-CF_3_ without a significant change in its colour (Fig. 2a and b). The fluorescence quantum yield (*Φ*_f_) of TAPP-CF_3_ moderately decreases with an increase in solvent polarity as previously observed for other TAPPs possessing electron-withdrawing groups at positions 2 and 5.^41,45^

Attaching pyren-1-yls to carbons 2 and 5 yields another A–D–A TAPP, *C*1-Pyr, that exhibits more pronounced bathochromic shifts and solvatofluorochromism than TAPP-CF_3_ (Fig. 2a). It is consistent with the facts that: (a) pyrene is a stronger electron acceptor than 1,3-bis(trifluoromethyl)benzene as evident from their reduction potentials^59,66^ and (b) pyrene is a larger substituent than 1,3-bis(trifluoromethyl)benzene, allowing stronger π-delocalization. In comparison with TAPP, *C*1-Pyr exhibits an 80 nm bathochromic shift in its absorption. Notably, absorption and emission of *C*1-Pyr is ≈70–80 nm hypsochromically shifted *vs.* that of 2,5-bis(perylen-1-yl)-1,4-dihydropyrrolo[3,2-*b*]pyrrole.^46^ The bathochromic shift of the fluorescence maxima is about 90 nm for non-polar media and increases to 120 nm with an increase in media polarity (Fig. 2a, Table 1). The colour of the fluorescence, however, stays overall green, shifting from bluish green for non-polar to yellowish green for polar media (Fig. 2b). These trends can originate from: (a) a significant extension of the π-conjugation over the pyrenes, and (b) an enhanced polarity of the emissive S_1_ state.

Unlike *Φ*_f_ of TAPP-CF_3_, *Φ*_f_ of *C*1-Pyr does not show a significant increase in non-polar media. While solvent polarity affects the energies of CT states, the extent of these effects depends on: (a) the size of delocalization of the hole and the electron on the donor and acceptor, respectively, and (b) on the donor–acceptor centre-to-centre distance. An increase in the size of the donor or the acceptor reduces the contributions from the solvation energy to the CT driving force.^67^ These differences are consistent with the theoretical results, and a comparison between the structures of TAPP-CF_3_ and *C*1-Pyr (Fig. 1) shows an increased donor–acceptor separation in the pyrene conjugate.

Linking pyren-1-yl substituents to the nitrogen atoms of the DHPP core at positions 1 and 4 leads to the emergence of uniquely different behaviour from that of the carbon-derivatized A–D–A TAPPs. The absorption spectrum of *N*1-Pyr/*t*Bu shows a distinct UV band between 300 and 400 nm that globally resembles that of TAPP, which is consistent with absorption of the central unit. Conversely, while this near UV region is where the pyrene substituents absorb, the *N*1-Pyr/*t*Bu spectra do not show the vibronic features from the S_0_ → S_2_ transitions characteristic for this PAH. That is, the electronic coupling through the nitrogen biaryl linkers is still strong enough to ensure an excited-state of mixed pyren-1-yls and the pyrrolopyrrole. These trends concur with an intermediate electronic-coupling regime.

The radiative deactivation of *N*1-Pyr/*t*Bu reveals a completely different behaviour than that of *C*1-Pyr. Variations of solvent polarity drastically change the colour of the *N*1-Pyr/*t*Bu fluorescence (Fig. 2b). The emission spectra of *N*1-Pyr/*t*Bu show bands with vibronic features between about 390 and 500 nm, along with broad featureless emission between 450 and 700 nm (Fig. 2a). A close look at the short-wavelength emission reveals that the bands between 390 and 500 nm are a superposition of pyrene and pyrrolopyrrole fluorescence with maxima practically invariant to solvent polarity (Fig. 2c).

Conversely, the broad featureless band shows pronounced solvatochromism and a decrease in amplitude with an increase in media polarity, *i.e.*, a CT like emission.

In contrast to previously described TAPPs with A–D–A architecture, *Φ*_f_ of *N*1-Pyr/*t*Bu is very small, even in toluene, and it further decreases with increasing solvent polarity. This behaviour is consistent with forming a CT state with well separated hole and electron exhibiting small rates of radiative deactivation. In addition to shifting the long-wavelength CT band, varying solvent polarity changes the ratios of the pyrene, pyrrolopyrrole, and CT contributions in the fluorescence spectra. This feature allows a remarkable white fluorescence to be obtained from *N*1-Pyr/*t*Bu when dissolved in solvents such as DMF (Fig. 2a), as a consequence of the spread of its emission from about 390 to 700 nm (Fig. 2a).

Increasing the electron-withdrawing character of the phenyl groups attached to carbons 2 and 5 weakens the electron-donating propensity of the pyrrolopyrrole core as in *N*1-Pyr/CF_3_ and *N*1-Pyr/CN (Fig. 1). The fluorescence quantum yields of in *N*1-Pyr/CF_3_ and *N*1-Pyr/CN are larger than those of *N*1-Pyr/*t*Bu (Table 1). Increasing solvent polarity decreases *Φ*_f_ of *N*1-Pyr/CF_3_ and *N*1-Pyr/CN similar to that of *N*1-Pyr/*t*Bu, which appears to originate mostly from the decrease in the relative intensity of their CT emission bands, indicating an enhancement of the preference for forming a dark CT state.^68^ These trends for the *N*-pyren-1-yl substituted TAPPs are consistent with a possible ES-SB behaviour.

The absorption spectra of *N*1-Pyr/CF_3_ and *N*1-Pyr/*t*Bu are similar and resemble those of TAPP-CF_3_ and TAPP, confirming photoexcitation from a ground-state localized on the pyrrolopyrrole cores. The fluorescence properties of *N*1-Pyr/CF_3_ and *N*1-Pyr/*t*Bu, on the other hand, are different. The emission spectra of *N*1-Pyr/CF_3_ show a single broad band only, exhibiting solvatochromism, reflecting their CT nature. The CT fluorescence of *N*1-Pyr/CF_3_ is less bathochromically shifted than that of *N*1-Pyr/*t*Bu (Fig. 2a), which is consistent with the presence of the trifluoromethyl electron-withdrawing groups. For toluene, the emission of *N*1-Pyr/CF_3_ shows a vibronic structure, similar to that of TAPP-CF_3_. Lowering media polarity, thus, appears to trigger fluorescence from states localized on the pyrrolopyrrole core. This can originate from raising the energy level of the CT state, bringing it close to that of the LE state of the pyrrolopyrrole. In such a case, conformational perturbation can trigger relaxation to, and emission from, the CT or the LE state. The CT emission of *N*1-Pyr/CF_3_ in toluene either overlaps with the band from the localized states or is completely quenched.

Placing CN groups on the *para*-position of the phenyls attached to carbons 2 and 5, further enhances the effects observed for *N*1-Pyr/CF_3_. For polar media, *e.g.*, DMF, *N*1-Pyr/CN shows a single fluorescence band with a relatively narrow peak. Lowering media polarity hypsochromically shifts the shoulder from the red spectral region and enhances the vibronic structure of the fluorescence band (Fig. 2a). Nitriles make the pyrrolopyrrole an even worse electron donor than the trifluoromethyls do and increase the energy level of the CT state close to that of the pyrrolopyrrole LE state even for polar solvents. While an increase in solvent polarity noticeably quenches the emission of the *N*-substituted pyren-1-yl TAPPs, the fluorescence of *N*1-Pyr/CN in DMF has a distinctly white appearance (Fig. 2b), *i.e.*, a remarkable feature related to the spread of the emission band from about 420 to 650 nm (Fig. 2a).

A distinct electronic feature of pyrene is the nodes of its HOMO and LUMO that extend from carbon 2 to carbon 7 (*N*1-Pyr/*t*Bu, Fig. 1).^65,69–74^ Therefore, moving the linkage position in the pyrenes from 1 to 2, further weakens the electronic coupling with the pyrrolopyrrole, as evident from the photophysical changes in *N*2-Pyr, in compassion with *N*1-Pyr/*t*Bu. The absorption of *N*2-Pyr appears as a superposition of the pyrrolopyrrole and the pyrene spectra. That is, the *N*2-Pyr absorption shows distinct narrow peaks at 325 and 341 nm on top of a broad UV band resembling the absorption of TAPP (Fig. 2a). The sharp bands at 325 nm and 341 nm closely resemble the vibronic features of the S_0_ → S_2_ pyrene absorption. The position of these sharp bands is invariant to media polarity, which agrees with the characteristics of S_0_ → S_2_ pyrene absorption. The S_0_ → S_1_ transition of this PAH, at around 370 nm, is forbidden and is barely noticeable in the spectra of derivatives with minimally perturbed electronic symmetry.^66^ These spectral features indicate that in *N*2-Pyr the pyrenes and the DHPP core act as separate chromophores, which perfectly concurs with remarkably weak electronic coupling between them.

Similar to *N*1-Pyr/*t*Bu, the fluorescence spectra of *N*2-Pyr show contributions from pyrene, pyrrolopyrrole, and CT fluorescence (Fig. 2a). Varying medium polarity induces changes in the colour of the *N*2-Pyr emission resembling those of *N*1-Pyr/*t*Bu (Fig. 2b). For each of the solvents, the CT emission maxima of *N*2-Pyr and *N*1-Pyr/*t*Bu are practically the same (Fig. 2a, Table 1). Despite the remarkably weak electronic coupling between the pyrene substituents and the pyrrolopyrrole core, the weak fluorescence *N*2-Pyr has a *Φ*_f_ comparable to the *Φ*_f_ of *N*1-Pyr/*t*Bu (Table 1). Thus, the difference in linking pyren-1-yl and pyren-2-yl substituents at the nitrogen atoms does not appear to have a noticeable impact on the excited-state dynamics of these TAPPs. Nevertheless, the bonding patterns with the pyrenes affects the electronic coupling in the ground state, as the differences in the absorption spectra of *N*1-Pyr/*t*Bu and *N*2-Pyr indicate.

Overall, decreasing the electronic coupling of the pyrrolopyrrole core with pyrenes by moving them from carbons 2 and 5 to nitrogens 1 and 4 affects the excited-state dynamics, but not the ground-state optical absorption. A further decrease in the electronic coupling by replacing pyren-1-yls with pyren-2-yls affects not only the fluorescence, but also the ground-state absorption of these TAPPs, *i.e.*, allowing the detection of optical transitions localized on the pyrenes.

### Theoretical analysis

Computational studies provide complementary insights into the nature of the excited states of the pyrene-conjugated TAPPs. We use time-dependent density-functional theory (TD-DFT) with the solvent effects modeled *via* the corrected-linear-response (cLR^2^) approach.^75^ This model provides a simultaneous description of both linear-response and state-specific solvent effects. We typically start from the DFT-optimized ground state considered in the *C*_*i*_ point group (a symmetry systematically checked with frequency calculation) and explore various excited states from there. See the ESI† for computational details and Table 2 for numerical results. In the many possible approaches used to represent excited states,^76^ we here use electron density difference (EDD) plots for their compactness.

**Table 2:** TD-DFT computed vertical transition wavelengths in toluene (black) and dimethylformamide (red), together with 0–0 wavelengths. We also report the dihedral angles

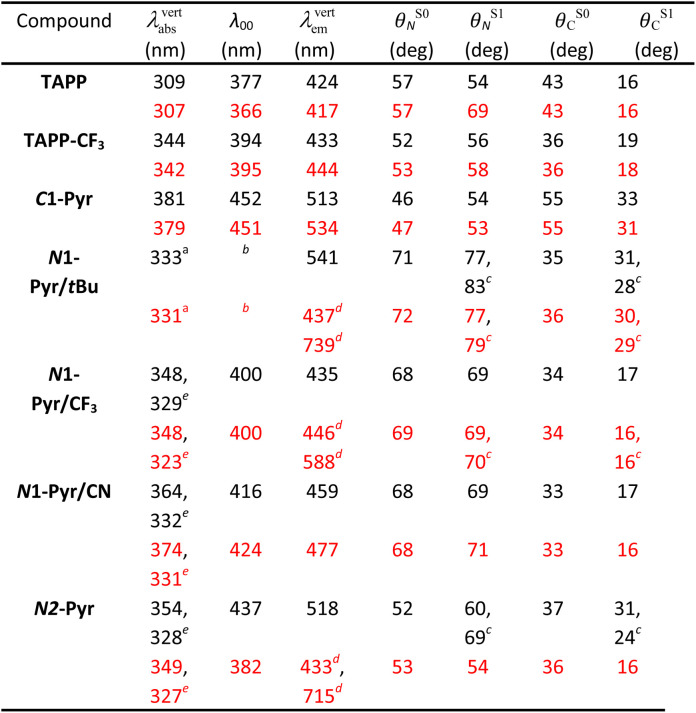

a Corresponding to the third state, largely dipole-allowed, see text.

b Cannot be directly computed since the main absorption and emission do not correspond to the same state.

c Two values due to ES-SB in the excited-state.

d Presence of both a local and a ES-SB state. Geometric and 0–0 data given for the former.

e Second (strongly) allowed absorption, see text.

For the model compounds, TAPP and TAPP-CF_3_, the calculations reveal a behaviour typical for pyrrolopyrrole derivatives with transitions to the lowest excited states that are strongly dipole-allowed (oscillator strength, *f*, larger than 1 in both molecules) and well separated from other transitions significantly contributing to the absorption. For these electronic transitions, the core acts as an electron donor and the *C*-substituents at the periphery – as acceptors. These compounds manifest a clear A–D–A character, as the electron-density-difference (EDD) plots reveal (Fig. S32 and S33†), and a significant increase in the electron density on the bond between the benzene rings and the pyrrolopyrrole core upon excitation. This translates into an increase in the planarity of the structure in the excited state, as the change in the dihedral angles show (Table 2). The presence of the CF_3_ group in TAPP-CF3 enhances this effect. The estimated amount of charge transferred is 0.55*e* and 0.62*e* in TAPP and TAPP-CF_3_, respectively. These trends are consistent with the experimental optical spectra described above.

Quantitatively, TD-DFT predicts rather negligible solvent effects, but a stronger bathochromic shift of the absorption (+35 nm) in comparison with the emission (+9 nm) when going from TAPP to TAPP-CF_3_. The latter compound presents a smaller Stokes' shift than the former. These trends perfectly match experimental findings. Comparing the experimentally estimated and theoretically calculated zero-to-zero wavelengths, *λ*_00_, which is a sound comparison,^77^ reveals small underestimations of the *λ*_00_ values by TD-DFT.

When attaching the pyrene to carbons 2 and 5, *i.e.*, *C*1-Pyr, TD-DFT predicts, as for TAPP and TAPP-CF_3_, two A–D–A vertical absorptions of *A*_u_ and *A*_g_ symmetries at 379 nm (*f* = 1.53) and 350 (*f* = 0.00) nm, respectively (see Fig. S34†). This moderate splitting (0.27 eV) is a signature of the significant coupling between the PP core and the pyrenes. For *C*1-Pyr, the calculations reveal significant bathochromic shifts of both absorption and emission, consistent with the experimental findings. For toluene, the theoretical *λ*_00_ is 452 nm which is close to the experimental value of 473 nm. The vertical fluorescence wavelength also undergoes a 21 nm bathochromic shift upon exchanging toluene solvating medium with DMF, which is not far from the experimental result of 31 nm. Despite the polarity-induced bathochromic shifts of the emission, theory foresees a stable *C*_*i*_ excited-state structure in DMF. Logically, the EDD plots show delocalization of the electron over the two pyrene units indicating an enhanced quadrupolar character, rather than a formation of a dipolar CT state (Fig. S34†).

**Fig. 3:**
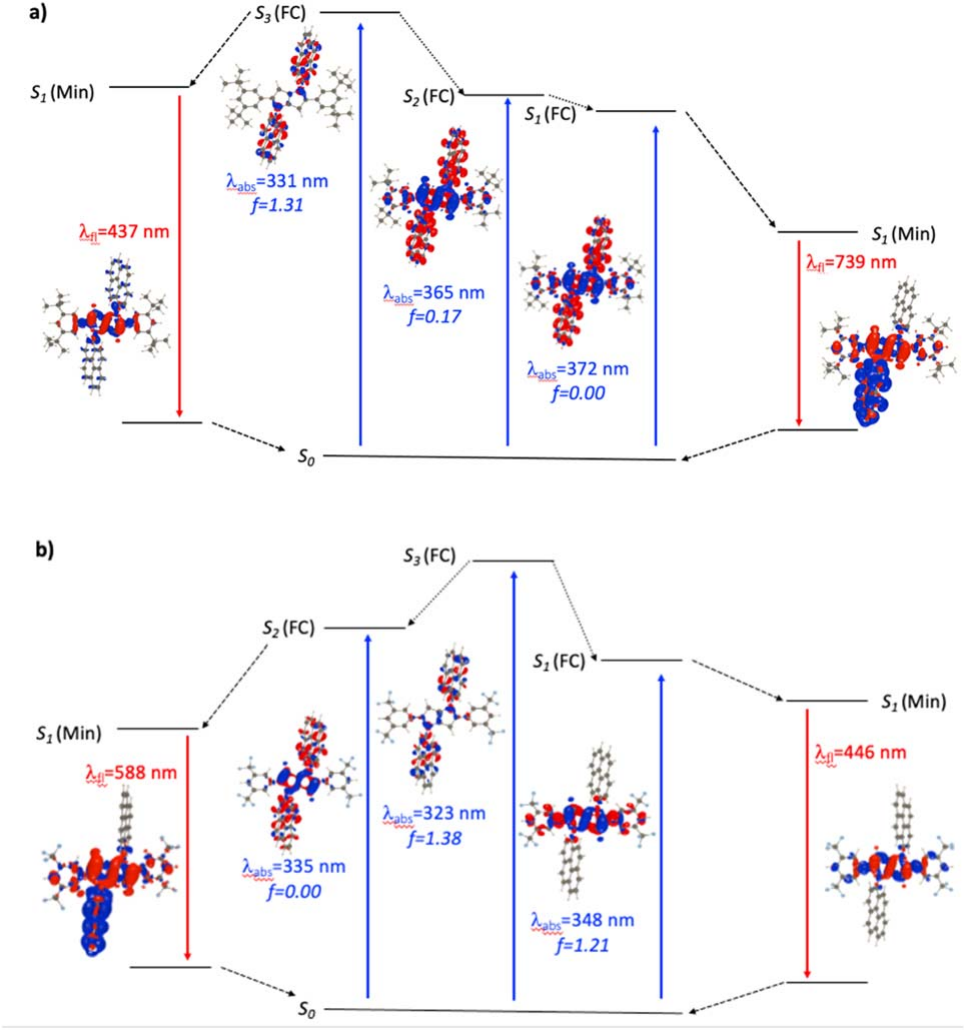
Jablonski-like diagram obtained with PCM(DMF)-TD-DFT for *N*1-Pyr/*t*Bu (a, top) and *N*1-Pyr/CF_3_ (b, bottom). For each transition, density difference plots (EDDs) in DMF are provided. Blue and red lobes correspond to decrease and increase of electron density upon transition. Contour: 1 × 10^−3^ a.u.

For the *N*1-Pyr derivatives, TD-DFT absorption calculations reveal transitions to several closely lying states with different characters and relative energies strongly affected by the substituents on the benzene rings attached to carbons 2 and 5. In *N*1-Pyr/*t*Bu, the transitions from S_0_ to the two lowest lying states correspond to absorption at 372 nm and 365 nm (Fig. 3a). For DMF, these transitions involve excitations to nearly degenerated states, *i.e.*, *A*_g_ (*f* = 0.00) and *A*_u_ (*f* = 0.17), the small splitting of 0.06 eV being consistent with a weak coupling between the DHPP core and the pyrene rings. The situation is similar in toluene. These two transitions (forbidden and weakly allowed) clearly correspond to hallmark CT from the PP core to the pyrenes. The third transition, at 331 nm, is strongly dipole-allowed (*f* = 1.31) and is localized mostly on the pyrenes, though the nitrogens of the pyrrolopyrrole still play a minor donating role (Fig. 3a). These features are consistent with the single-band absorption observed experimentally (Fig. 2a).

When optimizing the lowest excited state of *N*1-Pyr/*t*Bu in toluene, we note that enforcing the *C*_*i*_ point group induces the emergence of an imaginary frequency, which is a clear hint at ES-SB. The obtained minimum exhibits emission at 541 nm but with a low oscillator strength (*f* < 0.01), which is in a good agreement with the experimental feature of the low-intensity CT band appearing at 526 nm. Unsurprisingly, this effect is enhanced in DMF with a vertical emission at 739 nm (Fig. 3a, note the clear ES-SB for the emission). The computational results exaggerate the emission shift upon transitioning from toluene to DMF and the predicted spectral maximum for DMF is at a too long wavelength compared to experiment (610 nm). Nevertheless, the error on the emission wavelength remains acceptable for TD-DFT (<0.3 eV). Interestingly, the dipole moment of the emissive state attains a huge 23 D value for DMF, consistent with a strong solvatofluorochromic effect and a clear CT nature of the emissive state (Fig. 3a). The experimental emission seen at shorter wavelength (Fig. 2), seems to come a local excited state, and we located a possible candidate for this emission at 437 nm – this emission is centred on the DHPP in the calculation, whereas it seems to be pyrene-related experimentally.

In contrast, the absorption calculations for *N*1-Pyr/CF_3_ reveal a transition to a low-lying state that has the same nature as that of TAPP-CF_3_ (see Fig. 3b), *i.e.*, it is bright (*f* = 1.21), and is only very slightly red-shifted in comparison with TAPP-CF_3_. There are two close lying transitions of mixed contributions from the DHPP core and the two pyrenes: one bright (*A*_u_ symmetry, 323 nm) and one dark (*A*_g_ symmetry, 335 nm). The bright transition is blue-shifted by 25 nm compared to the longest-wavelength in *N*1-Pyr/*t*Bu band for DMF and has a similar intensity (*f* = 1.38). This evolution from *N*1-Pyr/*t*Bu to *N*1-Pyr/CF_3_ is consistent with the recorded steady-state spectra (Fig. 2a). For *N*1-Pyr/CF_3_, the optimization of the lowest excited state shows a stable *C*_*i*_ minimum in toluene. The radiative transition from a localized excited state is consistent with the measurements showing vibronic features in the emission, a small Stokes' shift and a rather large fluorescence quantum yield. In contrast, in DMF, TD-DFT indicates a small ES-SB for the lowest excited-state of *N*1-Pyr/CF_3_ (*i.e.*, the *C*_*i*_ minimum is not a stable minimum), but the computational analysis clearly underestimates the impact of this effect on the emission wavelength, as this transition remains mostly DHPP-centered (Fig. 3b). Starting the TD-DFT optimizations from either of the two higher excited states allowed us locating a clear (ES-SB) CT state with a computed emission at 588 nm, corresponding to the experimental value at 540 nm (Fig. 2).

In *N*1-Pyr/CN, the nitrile groups enhance the separation between the two types of states, *i.e.*, the pyrrolopyrrole-localized states are bathochromically shifted by 43 nm with respect to the CT one (Fig. S35†). This is consistent with the emergence of two separate bands in the steady-state absorption spectra of *N*1-Pyr/CN (Fig. 2a and c). For that compound, theory finds a stable symmetric excited-state structure in both toluene and DMF, and a standard emission is recovered (Fig. S35†), which obviously fits the measured spectra (Fig. 2a). In this system, the computed and measured 0–0 energies agree well.

In summary, the unexpected evolution of the fluorescence spectra in the *N*1-Pyr series originates from a fine interplay between CT and local excited states with relative positions of their energy levels depending on the substituents of the phenyls attached to carbons 2 and 5. In *N*1-Pyr/*t*Bu, the CT states are below the locally excited ones for polar and non-polar solvents. In *N*1-Pyr/CF_3_, the locally excited and the CT states are nearly degenerate. In *N*1-Pyr/CN, the energy level of the pyrene-pyrrolopyrrole CT state is elevated by the nitrile groups above the that of the lowest pyrrolopyrrole locally excited state.

**Fig. 4:**
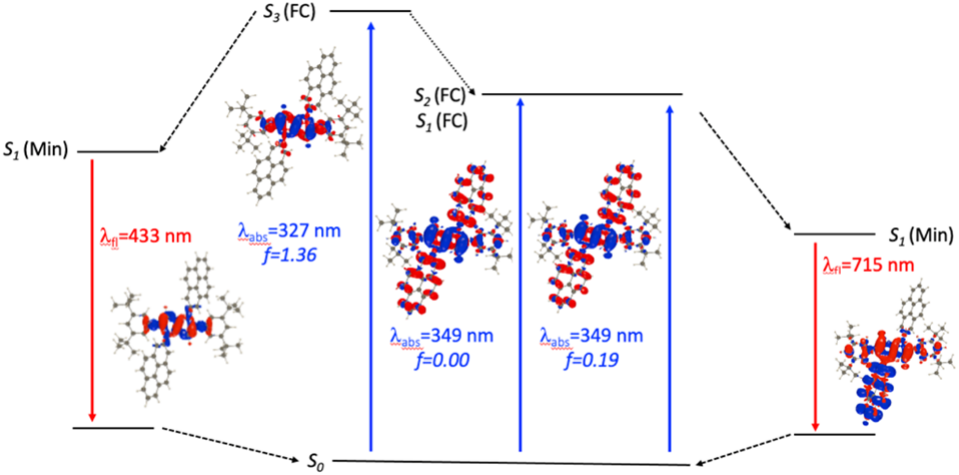
Jablonski-like diagram obtained with PCM(DMF)-TD-DFT for *N*2-Pyr. See caption of Fig. 3 for more details.

For *N*2-Pyr, the TD-DFT computes absorption to the lowest excited state at 349 nm for DMF and is slightly dipole-allowed (*f* = 0.19) and nearly perfectly degenerated with a dark state, a near-degeneracy indicating trifling coupling between the units (Fig. 4). These two states are results of pure CT transitions from the pyrrolopyrrole to one of the pyrenes, similarly to what we find in *N*1-Pyr/*t*Bu. The next bright absorption transition at 327 nm (*f* = 1.36) is a state localized on the pyrrolopyrrole, similar to the one of TAPP (Fig. 4). TD-DFT foresees the pyrene-centred excitation slightly higher in energy (*ca.* 10 nm, not shown in Fig. 4). In toluene, theory predicts a departure from symmetry for the *N*2-Pyr with CT emission at 518 nm, which agrees again with the measured spectra (Fig. 2a and c). Even more interesting is that the geometry optimization of the excited state of *N*2-Pyr in DMF, yields a stable minimum for the symmetric *C*_*i*_ structure with emission at 433 nm and 0–0 energy at 382 nm, both in line with the experimental data in that polar solvent. We could also locate an ES-SB CT structure for *N*2-Pyr in DMF showing a vertical (almost dark) emission at 715 nm, and the presence of two true minima (see EDD in Fig. 4), nicely correlates with the experimental results.

### Excited-state dynamics

Time-resolved spectroscopy allows probing the excited-state dynamics of the TAPP pyrene derivatives. Resorting to pump-probe transient-absorption (TA), we further sought to elucidate the mechanistic aspects of the excited-state processes and examine the limits of the analysis based on the information from the steady-state spectra and the theoretical predictions.

**Fig. 5:**
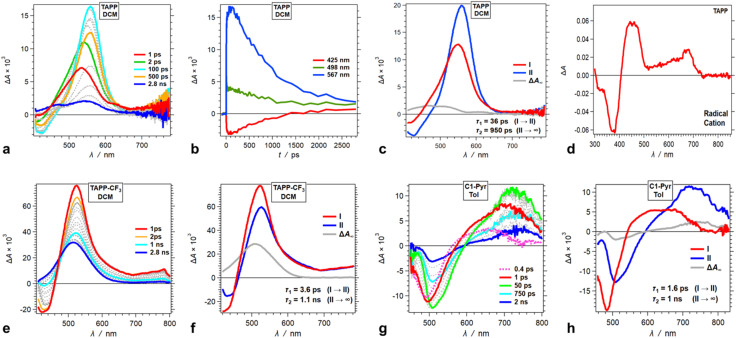
Transient absorption (TA) dynamics of (a–d) TAPP in DCM and its derivatives with substitutions at carbons 2 and 5, *i.e.*, (e and f) TAPP-CF_3_ in DCM and (g and h) *C*1-Pyr in toluene. (a, e and g) TA spectra from pump-probe measurements, *λ*_ex_ = 400 nm. (b) TA kinetic curves depicting rises and decays at selected wavelengths. (c, f and h) Evolution associated difference spectra (EADS) with the corresponding transition times constants, *τ*, obtained from global-fit (GF) analysis of the TA data. (d) Differential spectrum of the oxidized TAPP in DCM in the presence of 100 mM N(C_4_H_9_)_4_PF_6_, recorded at the peak of the first anodic wave using spectroelectrochemistry.

Upon photoexcitation, TAPP shows a distinct band at 560 nm, associated with the TA of its singlet excited state, S_1_ → S_1+*n*_, that undergoes a picosecond bathochromic shift and a rise, followed by a nanosecond decay (Fig. 5a). A negative signal at around 450 nm, ascribed to stimulated emission (SE), accompanies the TA bands. The picosecond rise (Fig. 5b and c) is consistent with relaxation of the Franck–Condon excited state, S_1_^(FC)^. The nanosecond decay agrees well with the lifetimes extracted from fluorescence decays of TAPP measured using time-correlated single-photon counting (TCSPC).

The TA spectra of the singlet excited state of TAPP-CF_3_ show a similar sharp absorption peak at around 520 nm with another broad small-amplitude band at around 750 nm. An SE signal at 420 nm accompanies these TA features, ascribed to the singlet excited state, ^1^TAPP-CF_3_* (Fig. 5e, S17a and g†). For DCM, these TA features show a small 4 ps decay with minute spectral shifts, followed by a nanosecond deactivation to a long-lived TA band at around 500 nm with no SE accompanying it (Fig. 5f).

Solvent polarity affects the rate and the Δ*A* amplitude of the initial picosecond TA decay ascribed to the relaxation of the S_1_^(FC)^ state. For toluene, the initial picosecond TA decay has a smaller amplitude (with no spectral shifts) and is 20 times slower than that for DCM (Fig. 5f and S17c†). For DMF, on the other hand, fast multiexponential picosecond decay decreases the amplitude of the ^1^TAPP-CF_3_* TA band by about a factor of three (Fig. S17i†).

For all solvents, the nanosecond decay of the ^1^TAPP-CF_3_* spectral signatures leads to a broad long-lived TA band at around 500 nm with no SE accompanying it (Fig. 5f, S17c and i†). The lifetime of this long-lived feature is outside the dynamic range of the pump-probe setup and its rise is consistent with triplet formation. An increase in solvent polarity substantially increases the amplitude of this triplet TA absorption (Fig. 5f, S17c and i†). Polar media tends to augment the extent of CS in dipolar and quadrupolar states, which in its turn can enhance the intersystem crossing to triplets. Consistent with the theoretical findings, however, the TA does not reveal evidence for possible formation a CT state from ES-SB of these A–D–A conjugates.

Going from TAPP-CF_3_ to *C*1-Pyr changes the appearance of the TA spectra but not the trends in the excited-state dynamics. Upon excitation, *C*1-Pyr displays a TA band in the red/NIR region with at around 700–800 nm. SE between 500 and 550 nm accompanies this NIR TA band (Fig. 5g), consistent with the S_1_ transient. These substantial shifts in the ^1^*C*1-Pyr* spectra, in comparison with those of ^1^TAPP-CF_3_* and ^1^TAPP*, indicate that pyrene moieties have a substantial effect on the S_1_ → S_*n*_ transitions.

Following some picosecond spectral changes, the ^1^*C*1-Pyr* undergoes nanosecond decay that dominates the TA dynamics (Fig. 5h). While an increase in solvent polarity tends to enhance the rates of the observed transformations, it does not alter the appearance of the TA spectral features (Fig. 5g and h, S19†). Most importantly, the TA spectra of *C*1-Pyr do not provide evidence for the formation of a CT state. The reduced pyrene acceptor, Py˙^−^, has a characteristic sharp absorption band at around 500 nm,^59^ and the oxidized pyrrolopyrrole acceptor, PP˙^+^, absorbs between 400 and 500 nm (Fig. 5d). None of these features emerges in the TA spectra of *C*1-Pyr, precluding claims for photoinduced CT and ES-SB, which agrees well with the theoretical findings for this pyrene-modified TAPP.

**Fig. 6:**
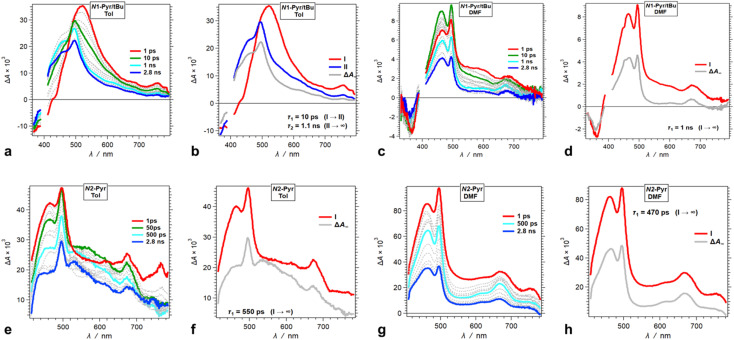
TA dynamics of TAPPs with pyrene substituents at nitrogens 1 and 4, *i.e.*, *N*1-Pyr/*t*Bu and *N*2-Pyr, for solvents with different polarity. (a, c, e and g) TA spectra from pump-probe measurements, *λ*_ex_ = 400 nm. (b, d, f and h) Evolution associated difference spectra (EADS) with the corresponding transition times constants, *τ*, obtained from global-fit (GF) analysis of the TA data. TA and transition spectra of (a and b) *N*1-Pyr/*t*Bu for toluene, (c and d) *N*1-Pyr/*t*Bu for DMF, (e and f) *N*2-Pyr for toluene, and (g and h) *N*2-Pyr for DMF.

Moving the pyrene substituents from carbons 2 and 5 to the nitrogens of the pyrrolopyrrole drastically alters the excited-state dynamics. Upon photoexcitation in non-polar media, *N*1-Pyr/*t*Bu displays a TA band at around 520 nm that closely resembles the absorption of the singlet excited-state transients of the model pyrrolopyrrole compounds (Fig. 6a and b). The 10 ps decay of this singlet TA leads to the emergence of broad spectral features comprising a sharp peak at 490 nm, reminiscent of the TA of the radical anion of pyrene, Py˙^−^,^59^ overlapping with the 450 nm TA band of the pyrrolopyrrole radical cation, PP˙^+^, that has a small-amplitude extension to 700 nm (Fig. 5d). This TA transformation is consistent with ES-SB with *k*_CT_ of 10^11^ s^−1^ and involves a transition from the TAPP symmetric singlet-excited state to a CT state with the hole on the pyrrolopyrrole and the electron on one of the pyrenes. It nicely concurs with the EDD plots (Fig. 4) showing the most stable excited-state structure that the computations predict for this compound.

An increase in solvent polarity accelerates the intramolecular CT leading to ES-SB. For DMF, in fact, we do not observe the formation of the TAPP singlet excited state. Instead, the spectral features of the CT species, Py˙^−^ and PP˙^+^, emerge within the 200 fs instrument-response time (Fig. 6c and d). For DMF, the spectra of the CT state exhibit well-defined sharp TA peaks of the two radical ions (Fig. 6c). For the medium- and low-polarity solvents, however, the CT TA peaks appear broad (Fig. 6a). This difference can originate from lessening the extent of charge separation and enhancing the delocalization of the spin-density distribution of the radical ions in low-polarity media. An interesting feature of the *N*1-Pyr/*t*Bu CT state is that after the nanosecond deactivation, its TA spectra do not appear to reach the zero baseline within the pump-probe dynamic range (Fig. 6b and d). This TA dynamics is consistent with forming a long-lived triplet CT state, *i.e.*, ^1^(PP˙^+^ − Py˙^−^) → ^3^(PP˙^+^ − Py˙^−^), which impedes the rates of charge recombination.^78–80^

Attaching the pyrene substituents *via* their 2-position to the pyrrolopyrrole nitrogens appears to enhance the CT kinetics. Photoexcitation of *N*2-Pyr leads to emergence of the TA signatures of the radical ions within the instrument-response time, not only for polar but also for non-polar solvation media (Fig. 6e–h). This finding appears as a surprise because among *N*2-Pyr, *N*1-Pyr/*t*Bu and *C*1-Pyr comprising the same donor and acceptors, ICT should be the fastest for *C*1-Pyr where the pyrene-pyrrolopyrrole electronic coupling is the largest, and slowest for *N*2-Pyr where the coupling through the pyrrolopyrrole nitrogens and the orbital node at position 2 of the pyrenes is the weakest. Nevertheless, the trends that TA spectroscopy and DFT provide are opposite, which warrants a close look at this train of thought and ask specifically, how does the electronic coupling affect the CT thermodynamics. That is, variations in the electronic coupling affect the electronic properties of the donor and the acceptors and alter the CT driving force. Such relatively small changes in the CT driving force can strongly affect the CT kinetics when the energy differences between the CT and locally excited states are small. The broad TA spectral features of *N*2-Pyr (and *N*1-Pyr/*t*Bu) in toluene suggests for species in their locally-excited, not only CT, states (Fig. 6e and f).

**Fig. 7:**
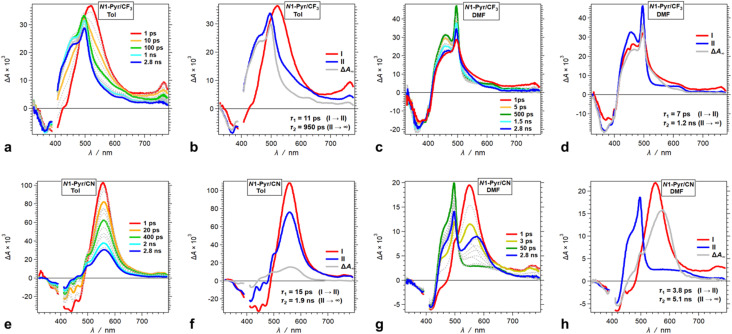
TA dynamics of TAPPs with pyren-1-yl substituents at nitrogens 1 and 4, and electron-withdrawing group attached to the phenyls at carbons 2 and 5, *i.e.*, *N*1-Pyr/CF_3_ and *N*1-Pyr/CN, for solvents with different polarity. (a, c, e and g) TA spectra from pump-probe measurements, *λ*_ex_ = 400 nm. (b, d, f and h) Evolution associated difference spectra (EADS) with the corresponding transition times constants, *τ*, obtained from global-fit (GF) analysis of the TA data. TA and transition spectra of (a and b) *N*1-Pyr/CF_3_ for toluene, (c and d) *N*1-Pyr/CF_3_ for DMF, (e and f) *N*1-Pyr/CN for toluene, and (g and h) *N*1-Pyr/CN for DMF.

Decreasing the electron-donating propensity of the pyrrolopyrrole core by placing 3,5-bis(CF_3_)C_6_H_3_ substituents on carbons 2 and 5, does not change the excited-state dynamics significantly. Similar to of *N*1-Pyr/*t*Bu, the TA of *N*1-Pyr/CF_3_ still shows picosecond formation of a CT state that undergoes small-amplitude changes to generate long-lived transients with the signatures of the radical ions, PP˙^+^ and Py˙^−^ (Fig. 7a–d). In polar media, such as DMF, the formation of the CT state of *N*1-Pyr/CF_3_ is within the instrument-response time (Fig. 7c and d), while for low-polarity solvents, the singlet-excited state of the pyrrolopyrrole forms initially (Fig. 7a and b). The emergence of the radical-ion TA spectra of *N*1-Pyr/*t*Bu and *N*1-Pyr/CF_3_ within the instrument-response time indicates: (1) direct excitation to the CT state; and/or (2) ICT leading to the ES-SB from an upper S_1_ state, *i.e.*, prior to the relaxation of S_1_^(FC)^. The small S_0_ → CT oscillator strength precludes the likelihood of the former.

The nitrile derivative, *N*1-Pyr/CN, has an even less electron-donating DHPP core than *N*1-Pyr/CF_3_. It makes the effects of solvent polarity on the ES-SB particularly pronounced. For polar media, such a DMF, 400 nm photoexcitation of *N*1-Pyr/CN generates its singlet excited state localized on the pyrrolopyrrole that transitions to a CT state with *k*_CT_ ∼3 × 10^11^ s^−1^ (Fig. 7g and h). This picosecond ES-SB of *N*1-Pyr/CN is slower than the femtosecond ES-SB of *N*1-Pyr/CF_3_ and *N*1-Pyr/*t*Bu in the same polar media.

For non-polar media, such as toluene, a band around 560 nm, associated with its singlet-excited state, dominates the TA of *N*1-Pyr/CN. It does not show signs of ES-SB, except the small shoulder at around 500 nm that can be reminiscent of the TA of Py˙^−^ (Fig. 7e). The nanosecond decay of the *N*1-Pyr/CN singlet leads to a long-lived transient consistent with a triplet that does not have a CT character (Fig. 7f). In fact, even the CT state that *N*1-Pyr/CN forms in DMF deactivates to a long-lived transient that exhibit a TA band characteristic of a triplet localized on the pyrrolopyrrole (Fig. 7h). Unlike *N*1-Pyr/*t*Bu and *N*1-Pyr/CF_3_, the energy level of the CT state of *N*1-Pyr/CN lies high enough not only to preclude accumulation of a CT triplet, ^3^(PP˙^+^ − Py˙^−^), but also to accommodate charge recombination leading to the pyrrolopyrrole triplet.

## Discussion

Attaching electron acceptors to carbons 2 and 5 of pyrrolopyrroles yields A–D–A structures that are prone to ES-SB.^45^ As a moderately good acceptor, however, pyrene does not have the same effect on the TAPP excited-state dynamics when attached to carbons 2 and 5 of TAPPs. Although *C*1-Pyr manifests solvatofluorochromism and a decrease in *Φ*_f_ in polar media, these phenomena do not originate from ES-SB. Rather, an increase in the extent of charge separation between the pyrrolopyrrole core and the two pyrenes enhances the polarity of the quadrupole emissive excited state and governs the observed photophysics of *C*1-Pyr.

Weakening the electronic coupling between the pyrene substituents and the pyrrolopyrrole core leads to the emergence not only of ES-SB, but also of optical absorption and emission transitions between states localized on the donor and the acceptors. For example, the distinct features of the ground-state pyrene absorption, *i.e.*, S_1_ → S_2_, emerge in the spectra of *N*2-Pyr (Fig. 2a). Furthermore, the fluorescence spectra of *N*1-Pyr and *N*2-Pyr show bands from the radiative deactivation of the pyrrolopyrrole and the pyrenes in addition to the CT emission from the state that ES-SB produces (Fig. 2a and c).

This behaviour of triple fluorescence from the CT and the LE states of the donor and the acceptors appears consistent with breaking the Kasha–Vavilov rule.^81^ The validity of this rule relies on the relatively fast relaxation of upper excited states to the lowest electronically excited state with the same multiplicity, *i.e.*, relaxation that is considerably faster than other processes that can originate from the upper states. Slow relaxation to the lowest excited state and/or fast competing processes from the upper states lead to “anti-Kasha” behaviour.^82,83^ Kasha–Vavilov rule, however, does not preclude the occurrence of the relatively slow processes from the upper states. Because of their relatively low probability, such slow processes usually have negligibly small and undetectable contributions to the observed photophysics.

**Fig. 8:**
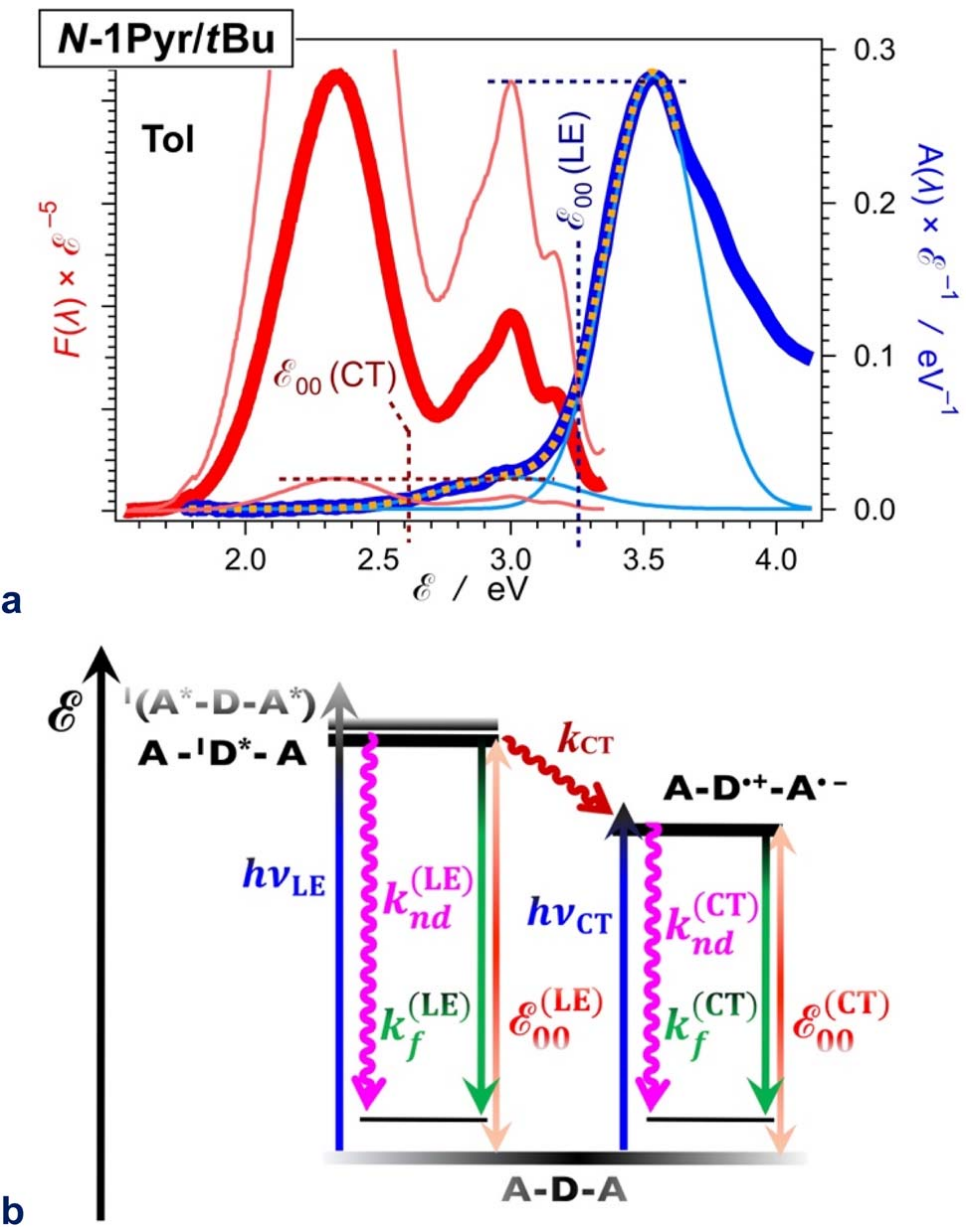
Excited-state dynamics of *N*1-Pyr/*t*Bu in toluene. (a) Extracting the zero-to-zero energies (*ε*_00_), *i.e.*, optical excitation energies, of the locally excited (LE) and the charge-transfer (CT) states from the steady-state fluorescence (red) and absorption (blue) spectra, plotted against energy scales after implementing transition-dipole-moment (TDM) corrections.^84^ The energy levels of the pyrene and pyrrolopyrrole lowest LE states are quite close (Fig. 2b). The overlapping S_0_ → CT and S_0_ → LE bands are deconvoluted by fitting the absorption spectrum to a sum of two Gaussian functions. Plotted are the data fit (orange dashed line) and the two Gaussian bands (thin blue lines). The fluorescence spectrum is normalized (the thin red lines) either to the CT or the LE absorption maximum as represented by the Gaussian curves, and the energy at which the normalized spectra cross is ascribed to *ε*_00_ : *ε*_00_^(LE)^ = 3.25 eV and *ε*_00_^(CT)^ = 2.61 eV. (b) Jablonski diagram constructed from experimental data. The energy of the radiative and non-radiative decays are estimated from the fluorescence maxima, *ε*_00_ are from the crossing points of the normalized spectra, and the CT driving force is estimated from *ε*_00_, *i.e.*, Δ*G*_CT_^(0)^ = *ε*_00_^(CT)^ − *ε*_00_^(LE)^ = 0.64 eV. For pyrrolopyrrole, *k*_f_^(LE)^ = 4.4 × 10^8^ s^−1^ and *k*_nd_^(LE)^ = 5.7 × 109 s^−1^; *k*_f_^(CT)^ = 1.9 × 10^7^ s^−1^ and *k*_nd_^(CT)^ = 2.9 × 10^8^ s^−1^; *k*_CT_ = 1.0 × 10^11^ s^−1^.

The *N*1-Pyr and *N*2-Pyr conjugates have weakly fluorescent CT states. Their radiative-decay rate constants, *k*_f_^(CT)^, are about one-to-three orders of magnitude smaller than those for the LE states, *k*_f_^(LE)^, as observed for TAPP and TAPP-CF_3_, for instance (Table 1). These small values of *k*_f_^(CT)^ are consistent with the weak donor–acceptor electronic coupling. As a result, the CT fluorescence of these conjugates is weak enough to allow the observation of the small emission signals from the LE states (Fig. 2a and c, 8a).

For example, *N*1-Pyr/*t*Bu in toluene shows triple fluorescence (Fig. 2a and 8a) with total *Φ*_f_ of about 0.06 (Table 1). The fluorescence from the pyrrolopyrrole LE state amounts to about 10% of the total integrated emission, suggesting that *Φ*_f_^(LE)^ of the donor in *N*1-Pyr/*t*Bu is about 6 × 10^−3^. The LE states undergo their inherent radiative and non-radiative deactivation to the ground state with rate constants *k*_f_^(LE)^ and *k*_nd_^(LE)^, respectively (Fig. 8b). The values of *k*_f_ and *k*_nd_ of TAPP (Table 1) provide good estimates for *k*_f_^(LE)^ and *k*_nd_^(LE)^ of the pyrrolopyrrole LE state. The picosecond CT dominates the deactivation of the LE states, with *k*_CT_ of 10^11^ s^−1^ (Fig. 6b and 8b). The fluorescence quantum yield of the pyrrolopyrrole in *N*1-Pyr/*t*Bu, thus, can be approximated to the ratio of *k*_f_^(LE)^ and *k*_CT_. The kinetic model (Fig. 8b), therefore, yields *Φ*_f_^(LE)^ ≈ *k*_f_^(LE)^*k*_CT_^−1^ ≈ 4.4 × 10^−3^, which agrees well with the *Φ*_f_^(LE)^ of 6 × 10^−3^ for pyrrolopyrrole in *N*1-Pyr/*t*Bu estimated from steady-state spectra. Applying broadly this train of thought to all *N*1-Pyr and *N*2-Pyr conjugates, for solvents where they show CT and LE fluorescence, reveals how slowing down the radiative deactivation of the lowest electronically excited state, *i.e.*, *k*_f_^(CT)^ in this case (Fig. 8b), can lead to anti-Kasha photoemission behaviour showing the inherently weak fluorescence from upper electronically excited states in the steady-state spectra.

While compromising the adiabatic nature of the ICT, weakening the pyrene-pyrrolopyrrole electronic coupling enhances the rates of CT leading to ES-SB. This trend may appear counterintuitive. Assuming that the driving force and the reorganization energy for the symmetry-breaking CT are almost the same for *N*2-Pyr, *N*1-Pyr/*t*Bu and *C*1-Pyr, ensures the same Franck–Condon contributions to the ICT kinetics for the three conjugates. That is, strengthening the donor–acceptor electronic coupling should increase the CT rates. The TA results, however, reveal the opposite trend, which warrants examination of the above assumption.

Strengthening the electronic coupling perturbs the properties of the donor and the acceptor, including their excitation energy and reduction potentials, which affects the CT driving force. In *C*1-Pyr, a decrease in the reduction potential of pyrene can originate from its strong coupling with the electron-rich pyrrolopyrrole. That is, strong coupling worsens the capability of the pyrenes to act as electron acceptors. Concurrently, the strong coupling with two pyrenes increases the reduction potential of the oxidized pyrrolopyrrole core making it a worse electron donor. Furthermore, expanding the π-conjugation to the well coupled PAH substituents, lowers the zero-to-zero photoexcitation energy, *ε*_00_, of *C*1-Pyr. Therefore, the strong electronic coupling in *C*1-Pyr elevates the energy level of its CT state, while decreasing *ε*_00_, and overall depletes the CT driving force. These trends are consistent with the lack of TA and theoretical evidence for CT-driven ES-SB.

At the other side of the spectrum, the donor–acceptor electronic coupling in *N*2-Pyr is weak enough not only to preclude the emergence of a CT band in the ground-state absorption, but also to prevent perturbation of the reduction potentials of the pyrene and the oxidized pyrrolopyrrole, ensuring sufficiently large CT driving forces. Concurrently, the electronic coupling is strong enough for *N*2-Pyr to mediate sub-picosecond CT that drives ES-SB even in non-polar solvents.

As an intermediate case, *N*1-Pyr/*t*Bu mediates CT leading to ES-SB unlike *C*1-Pyr. Nevertheless, the picosecond CT in the photoexcited *N*1-Pyr/*t*Bu is slower than that in *N*2-Pyr, even though, the donor–acceptor electronic coupling is weaker in the latter. Therefore, the effects of electronic coupling on the CT driving forces appears to have a dominating impact on the ICT kinetics.

The ES-SB involves CT that produces well separated radical ions localized on the donor and on one of the acceptors. The relaxation of the FC excited states of TAPPs decreases the dihedral angles *θ*_C_ between the pyrrolopyrrole core and the substituents at carbons 2 and 5 (Table 2). The dihedral angles with the nitrogen substituents, *θ*_N_, are minimally perturbed or undergo some increase. This planarization at the *C*-substituents enhances the donor–acceptor electronic coupling in *C*1-Pyr and decreases the propensity for formation of a CT excited state with localized radical ions, precluding ES-SB. That is, strongly coupled A–D–A systems are prone to sustain the quadrupole symmetry in their excited states. Weakly coupled A–D–A conjugates, on the other hand, have a tendency to form CT states with localized radical ions, necessary for ES-SB. Hence, the extent of localization of the radical ions, governed by the electronic coupling in the excited state, offers an alternative consideration for the likelihood of ES-SB to occur.

Overall, favourable CT driving forces, affected by the electronic coupling in these biaryl-linked A–D–A conjugates, are the most important requirement for ES-SB. Even diabatic CT through weakly-coupling biaryl linkers is fast enough to ensure ES-SB within the lifetime of the symmetric excited states. The effects on the CT rates that the trifluoromethyl and nitrile groups exert in *N*1-Pyr/CF_3_ and *N*1-Pyr/CN, further supports the governing role of the CT thermodynamics for the ES-SB of these TAPPs.

## Conclusions

Varying the donor–acceptor electronic coupling through pyrene-pyrrolopyrrole biaryl linkers of TAPP conjugates allows the emergence of some unprecedented CT phenomena. A decrease in the electronic coupling not only enhances the CT rates driving ES-SB, but also leads to conditions of white fluorescence. For almost a century, the Born–Oppenheimer (BO) approximation has allowed separation of the analysis of the electronic and nuclear contributions to transition rates.^85^ The thermodynamic driving force, along with the reorganization energy, provides a means for characterizing the nuclear contribution to the rate of a diabatic process.^31^ Enhancing donor–acceptor electronic coupling affects not only the kinetics, but also the thermodynamics of CT processes. The latter, in its own turn, also affects the former. While this finding does not contradict the BO approximation, it warrants caution in the thermodynamic analysis of CT and other processes where adiabaticity may prevail. Such considerations impact energy science, along with photonics and electronics engineering.

## Author contributions

Conceptualization: J. A. C. and D. T. G.; investigation: J. A. C., D. K., O. V., M. K., K. J. K., B. K. and D. J.; supervision: D. T. G., D. J. and V. I. V.; visualization: M. K., J. A. C.; calculation: D. J. writing – original draft: J. A. C., V. I. V., D. J.; D. T. G.; writing – review & editing: V. I. V., D. J.; D. T. G. All the authors discussed the results and commented on the manuscript.

## Conflicts of interest

There are no conflicts to declare.

## Supplementary Material

SC-014-D3SC03812B-s001
