# Supplementary material for: The magic of biaryl linkers: the electronic coupling through them defines the propensity for excited-state symmetry breaking in quadrupolar acceptor–donor–acceptor fluorophores
Source: Chem Sci. 2023 Nov 6;14(46):13537–50. doi: 10.1039/d3sc03812b (PMC10685337; doi:10.1039/d3sc03812b)
Supplement: SC-014-D3SC03812B-s001 [file SC-014-D3SC03812B-s001.pdf]

Electronic Supplementary Material (ESI) for Chemical Science.
This journal is © The Royal Society of Chemistry 2023

Electronic Supplementary Information

The magic of biaryl linkers: The electronic coupling through them defines the propensity for excited-state symmetry breaking in quadrupolar acceptor-donor-acceptor fluorophores

John A. Clark,[a] Damian Kusy,[b] Olena Vakuliuk,[b] Maciej Krzeszewski,[b] Krzysztof J. Kochanowski,[b] Beata Koszarna,[b] Omar O'Mari,[a] Denis Jacquemin,*[c,d] Daniel T. Gryko*[b] and Valentine I. Vullev*[a,e,f,g]

Table of Contents

Materials……………………………………………………………………………………S2

NMR Results…………………………………………………………………………………S5

Methods……………………………………………………………………………………..S18

Spectroscopy of TAPP……………………………………………………………………S19

Spectroscopy of TAPP-CF_3………………………………………………………………S20

Spectroscopy of *C*1-Pyr……………………………………………………………………S21

Spectroscopy of *N*1-Pyr/*t*Bu ………………………………………………………………S22

Spectroscopy of *N*2-Pyr ……………………………………………………………………S23

Spectroscopy of *N*1-Pyr/CF_3……………………………………………………………S24

Spectroscopy of *N*1-Pyr/CN…………………………………………………………………S25

Dependence of the emission on the excitation wavelength……………………………..S26

Electrochemistry and spectroelectrochemistry..…………………………………………S28

Theoretical analysis……………………………………………………………………………S29

Materials

General procedure for the synthesis of 1,2,4,5-tetraarylo-1,4-dihydropyrrolo[3,2-*b*]pyrroles: Parent aldehyde (2 eq.) and aniline (2 eq.) were dissolved in a AcOH/toluene or AcOH/DCE mixture and reacted at 50 °C for 1 h. After that time, $Fe(ClO_4)_3 \cdot x\ H_2O$ (6 mol%) and diacetyl (**XX**) (1 eq.) were added. The resulting mixture was stirred at 50 °C (oil bath) in an open flask under air overnight. Unless otherwise noted, crude TAPP was filtered off, washed with cold methanol or acetonitrile and dried under vacuum.

1,2,4,5-tetrakis(3,5-di-*tert*-butylphenyl)-1,4-dihydropyrrolo[3,2-b]pyrrole (**TAPP**):

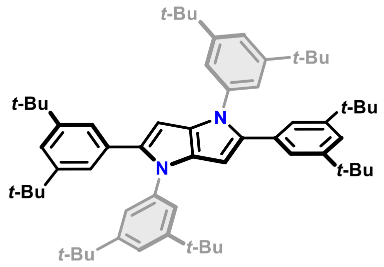

Following the general procedure, **TAPP** was prepared from 3,5-di-*tert*-butylbenzaldehyde (**XX**) (532 mg, 2.4 mmol), 3,5-di-*tert*-butylaniline (**XX**) (500 mg, 2.4 mmol) and diacetyl (**XX**) (107 µL, 1.2 mmol) in glacial acetic acid / toluene mixture (8 ml, 1:1 v/v). The precipitate was filtered off, washed with methanol. The precipitate was purified by a column chromatography (SiO_2, hexane : ethyl acetate = 9:1) and -pseudo-recrystallized from methanol. Drying under vacuum afforded **TAPP (**200 mg, 19%) as a white solid. M.p. = 322–323 °C, R_f= 0.18 (SiO_2, hexane). **^{1}H NMR (600 MHz, $CDCl_3$)** δ 7.21 (t, *J* = 1.8 Hz, 2H), 7.20 (t, *J* = 1.9 Hz, 2H), 7.07 (d, *J* = 1.8 Hz, 4H), 7.04 (d, *J* = 1.9 Hz, 4H), 6.41 (s, 2H), 1.19 (s, 36H), 1.17 (s, 36H). **^{13}C NMR (151 MHz, $CDCl_3$)** δ 151.6, 150.4, 139.4, 137.1, 133.4, 130.5, 123.8, 120.6, 120.2, 119.1, 92.9, 76.9, 34.9, 34.8, 31.47, 31.45. **HRMS (EI)** calcd for $C_{62}H_{86}N_2$: 858.6786 $[M]^{\cdot+}$, found: 858.6790.

2,5-bis(3,5-bis(trifluoromethyl)phenyl)-1,4-bis(3,5-di-*tert*-butylphenyl)-1,4-dihydropyrrolo[3,2-b]pyrrole (**TAPP-CF$_3$**):

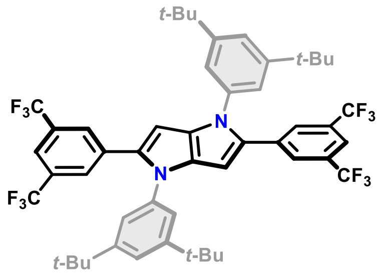

Following the general procedure, **TAPP-CF$_3$** was prepared from 3,5-di-*tert*-butylbenzaldehyde (**XX**) (1.18 g, 4.9 mmol), 3,5-bis(trifluoromethyl)aniline (**XX**) (1.0 g, 4.9 mmol) and diacetyl (**XX**) (214 µL, 2.43 mmol) in glacial acetic acid / toluene mixture (8 ml, 1:1 v/v). The filtrate was purified by a column chromatography (SiO_2, hexane : ethyl acetate = 9:1) and pseudo-recrystallized from methanol. Drying under vacuum afforded **TAPP-CF$_3$ (**130 mg, 6%) as a white solid. M.p. = 296–297 °C, R_f= 0.50 (SiO_2, hexane). **^{1}H NMR (500 MHz, $CDCl_3$)** δ 7.58 (bs, 2H), 7.54 (bs, 4H), 7.40 (t, *J* = 1.7 Hz, 2H), 7.08 (d, *J* = 1.7 Hz, 4H), 6.51 (s, 2H), 1.25 (s, 36H). **^{13}C NMR (126 MHz, $CDCl_3$)** δ 152.9, 138.2, 135.6, 134.2, 132.8, 131.5 (q, *J* = 33.3 Hz), 127.6, 124.41 (q, *J* = 273 Hz), 121.2, 120.6, 119.2 (d, *J* = 3.8 Hz), 94.7, 35.1, 31.3. **HRMS (EI)** calcd for $C_{50}H_{50}N_2F_{12}$: 906.3777 $[M]^{\cdot+}$, found: 906.3801.

2,5-bis(3,5-di-*tert*-butylphenyl)-1,4-di(pyren-1-yl)-1,4-dihydropyrrolo[3,2-b]pyrrole (*N*1-Pyr/*t*Bu): Following the general procedure, ***N*1-Pyr/*t*Bu** was prepared from 3,5-di-*tert*-butylbenzaldehyde (**XX**) (1.0 g, 4.6 mmol) 1-aminopyrene (**XX**) (1.0 g, 4.6 mmol) and diacetyl (**XX**) (138 µL, 2.3 mmol) in glacial acetic acid / 1,2- dichlorobenzene mixture (5 ml, 1:1 v/v). The precipitate was filtered off, washed with methanol. The precipitate was purified by a column chromatography (SiO_2, hexane : ethyl acetate = 9:1) and pseudo-recrystallized from methanol. Drying under vacuum afforded ***N*1-Pyr/*t*Bu (**200 mg, 5%) as a yellowish solid. M.p. = >350 °C (decomp.) R_f= 0.30, (SiO_2, hexane/CH_2Cl_2, 4:1). **^{1}H NMR (500 MHz, $CDCl_3$)** Mixture of atropoisomers (ratio: ~1:0.45) δ 8.50 (d, *J* = 9.2 Hz, 2H), 8.28 – 8.25 (m, 6H), 8.24 – 8.21 (m, 1H), 8.18 (d, *J* = 9.4 Hz, 3H), 8.16 – 8.05 (m, 11H), 8.06 – 7.99 (m, 2H), 7.76 (d, *J* = 8.0 Hz, 2H), 6.97 (t, *J* = 1.8 Hz, 2H), 6.93 (d, *J* = 1.8 Hz, 1.5H), 6.91 (d, *J* = 1.8 Hz, 4H), 6.35 (s, 0.8H), 6.32 (s, 2H), 0.85 (s, 36H), 0.84 (s, 14H). **^{13}C NMR (126 MHz, $CDCl_3$)** δ 150.3, 150.2, 139.2, 135.11, 135.07, 133.9, 133.8, 132.4, 131.5, 131.4, 131.33, 131.26, 130.4, 128.5, 128.2, 127.65, 127.62, 127.4, 127.3, 126.8, 126.6, 126.4, 126.3, 125.7, 125.6, 125.51, 125.48, 125.42, 125.36, 125.2, 124.9, 124.7, 123.64, 123.59, 122.1, 119.7, 119.6, 94.2, 34.6, 31.1. **HRMS (EI)** calcd for $C_{66}H_{62}N_2$: 882.4908 $[M]^{\cdot+}$, found: 882.4932.

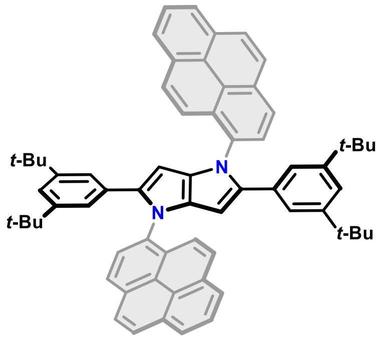

1,4-di(pyrene-1-yl)-2,5-bis(3,5-di-(trifluoromethyl)phenyl)-1,4-dihydropyrrolo[3,2-*b*]pyrrole (*N*1-Pyr/CF_3): Following the general procedure, ***N*1-Pyr/CF_3** was prepared from 3,5-di-(trifluoromethyl)benzaldehyde (**XX**) (332 µL, 2.0 mmol) 1-aminopyrene (**XX**) (434.0 mg, 2.0 mmol) and diacetyl (**XX**) (88 µL, 1.0 mmol) in glacial acetic acid / DCE mixture (8 ml, 1:3 v/v). The precipitate was filtered off, washed with methanol and pseudo-recrystallized from acetonitrile. Drying under vacuum afforded ***N*1-Pyr/CF_3 (**146 mg, 6%) as an off-white solid (mixture of atropisomers). M.p. = 329–330 °C. R_f= 0.46 (SiO_2, hexane/CH_2Cl_2, 4:1). **^{1}H NMR (500 MHz, $CDCl_3$)** δ 8.32 – 8.22 (m, 6H), 8.18 (m, 4H), 8.14 – 8.07 (m, 4H), 8.03 (d, *J* = 8.0 Hz, 2H), 7.99 (d, *J* = 9.2 Hz, 2H), 7.48 (bs, 4H), 7.37 (bs, 2H), 6.46 (bs, 2H). **^{13}C NMR (126 MHz, $CDCl_3$, $J_{C\text{-}F}$-coupling constants can't be identified due to the complexity of spectra)** δ 136.5, 136.4, 135.1, 135.04,135.03 134.9, 132.5, 132.4, 131.5-130.9 (m), 129.6 (m), 129.5, 129.27, 129.22, 128.4, 128.3, 127.7, 127.4, 127.2, 127.1, 126.6 (m), 126.1, 126.0, 125.9, 125.84, 125.80, 125.62, 124.57, 124.49, 124.0, 122.1, 122.0, 121.9, 119.4, 119.1 (m), 95.9, 95.4. **HRMS (EI)** calcd for $C_{54}H_{26}N_2F_{12}$: 930.1899 $[M]^{\cdot+}$, found: 930.1918.

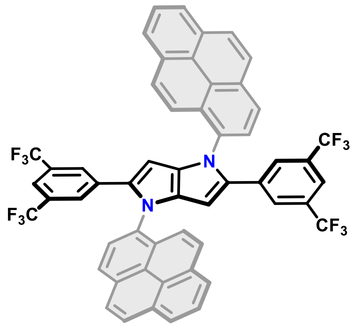

2,5-bis(3,5-di-*tert*-butylphenyl)-1,4-di(pyren-2-yl)-1,4-dihydropyrrolo[3,2-b]pyrrole (***N*2-Pyr**): Following the general procedure, ***N*2-Pyr** was prepared from 3,5-di-*tert*-butylbenzaldehyde (**XX**) (140.0 mg, 0.64 mmol) 2-aminopyrene (**XX**) (140.0 mg, 0.64 mmol) and diacetyl (**XX**) (28.3 µL, 0.32 mmol) in glacial acetic acid / 1,2- dichlorobenzene mixture (5 ml, 1:1 v/v). The precipitate was filtered off, washed with methanol. The precipitate was purified by a column chromatography (SiO_2, hexane : ethyl acetate = 9:1) and pseudo-recrystallized from methanol. The precipitate was filtered off and washed with

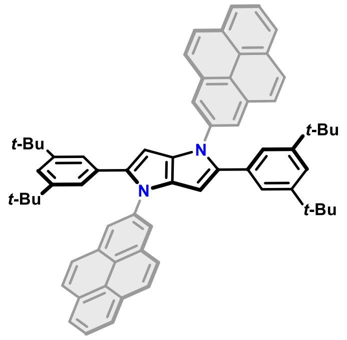

methanol. Then, the solvent was removed and the residue was loaded onto the column and chromatographed (SiO_2, hexane : ethyl acetate = 9:1). Drying under vacuum afforded ***N*2-Pyr** (66 mg, 23%) as a yellowish solid. M.p. = >350 °C (decomp.) R_f= 0.27 (SiO_2, hexane/CH_2Cl_2, 4:1). **^{1}H NMR (500 MHz, $CDCl_3$)** δ 8.21 (d, *J* = 7.7 Hz, 4H), 8.15 (s, 4H), 8.09 (d, *J* = 8.9 Hz, 4H), 8.02 (t, *J* = 7.7 Hz, 2H), 7.97 (d, *J* = 8.9 Hz, 4H), 7.19 (bs, 2H), 7.12 (bs, 4H), 6.71 (s, 2H), 1.03 (s, 36H). **^{13}C NMR (126 MHz, $CDCl_3$)** δ 150.6, 138.3, 137.4, 132.6, 132.0, 131.9, 131.1, 128.2, 127.3, 125.9, 125.5, 124.6, 123.2, 122.6, 121.7, 120.3, 94.6, 34.8, 31.3. **HRMS (EI)** calcd for $C_{66}H_{62}N_2$: 882.4908 $[M]^{\cdot+}$, found: 882.4873.

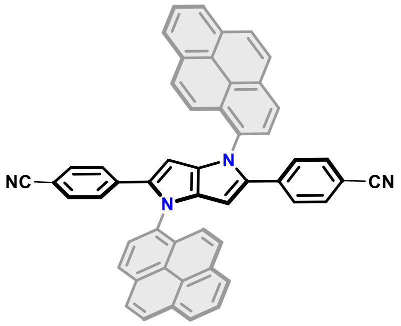

1,4-di(pyrene-1-yl)-2,5-di(4-cyanophenyl)-1,4-dihydropyrrolo[3,2-*b*]pyrrole (***N*1-Pyr/CN**):

Following the general procedure, ***N*1-Pyr/CN** was prepared from 4-cyanobenzaldehyde (**XX**) (262.0 mg, 2.0 mmol), 1-aminopyrene (**XX**) (434.0 mg, 2.0 mmol) and diacetyl (**XX**) (88 µL, 1.0 mmol) in glacial acetic acid / DCE mixture (8 ml, 1:3 v/v). The precipitate was filtered off and washed with MeOH acetonitrile. The filtrate was pseudo-recrystallized from hot THF and dried under vacuum to afford ***N*1-Pyr/CN** (212 mg, 30%) as a yellow solid (mixture of atropoisomers, in 10:9 ratio). M.p. = >350 °C (decomp.). R_f= 0.6 (SiO_2, hexane/CH_2Cl_2, 1:2). **^{1}H NMR (500 MHz, DMSO-*d*$_6$)** δ 8.48 – 8.29 (m, 11H), 8.25 (d, *J* = 9.3 Hz, 1H), 8.22 – 8.14 (m, 4H), 7.95 – 7.88 (m, 2H), 7.42-7.39 (m, 4H), 7.24-7.16 (m, 4H), 6.64, 6.62 (2 x s, 2H). **^{13}C NMR** was not recorded due to the low solubility of the compound. **HRMS (EI)** calcd for $C_{52}H_{28}N_4$: 708.2308 $[M]^{\cdot+}$, found: 708.2299.

2,5-di(pyren-1-yl)-1,4-di(4-octadecylphenyl)-1,4-dihydropyrrolo[3,2-b]pyrrole (***C*1-Pyr**):

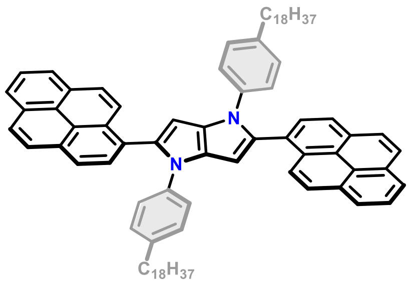

Following the general procedure, ***C*1-Pyr** was prepared from 1-pyrenecarboxaldehyde (**XX**) (460 mg, 2.0 mmol), 4-octadecylaniline (**XX**) (690 mg, 2.0 mmol) and diacetyl (**XX**) (88 µL, 1.0 mmol) in glacial acetic acid/1,2-dichlorobenzene mixture (3 ml, 1:1 v/v). The precipitate was filtered off, washed with CH_3CN and MeOH. The precipitate was purified by pseudo-recrystallization from AcOEt and recrystallization from toluene. Drying under vacuum afforded ***C*1-Pyr** (445 mg, 38%) as a yellowish solid. M.p. = 181-182 °C. R_f = 0.54 (SiO_2, hexane/CH_2Cl_2, 7:1). **^{1}H NMR (500 MHz, toluene-*d*$_8$, 80°C)** δ 8.61 (d, *J* = 9.1 Hz, 2H), 7.98 (d, *J*=7.8, 2H), 7.91-7.81 (m, 6H), 7.80-7.74 (m, 6H), 7.70 (t, *J* = 7.6 Hz, 2H), 7.27 (d, *J* = 8.2 Hz, 4H), 6.83 (s, 2H), 6.75 (d, *J* = 8.1 Hz, 4H), 2.26 (t, *J* = 7.6 Hz, 4H), 1.61 – 1.21 (m, 54H), 1.18-1.10 (m, 10H), 0.87 (t, *J* = 6.5 Hz, 6H). **^{13}C NMR (126 MHz, toluene-*d*$_8$, 80°C)** δ 149.1, 147.8, 143.5, 141.3, 141.2, 140.8, 140.2, 139.7, 139.3, 138.7, 138.2, 137.3, 136.7, 135.4, 135.1, 134.9, 134.7, 134.3, 133.8, 133.6, 107.7, 44.6, 41.3, 40.3, 39.2, 39.1, 39.1, 39.0, 38.8, 38.8, 38.6, 32.0, 23.1. **HRMS (APCI)** calcd for $C_{86}H_{103}N_2$: 1163.8116 $[M+H]^+$, found: 1163.8069.

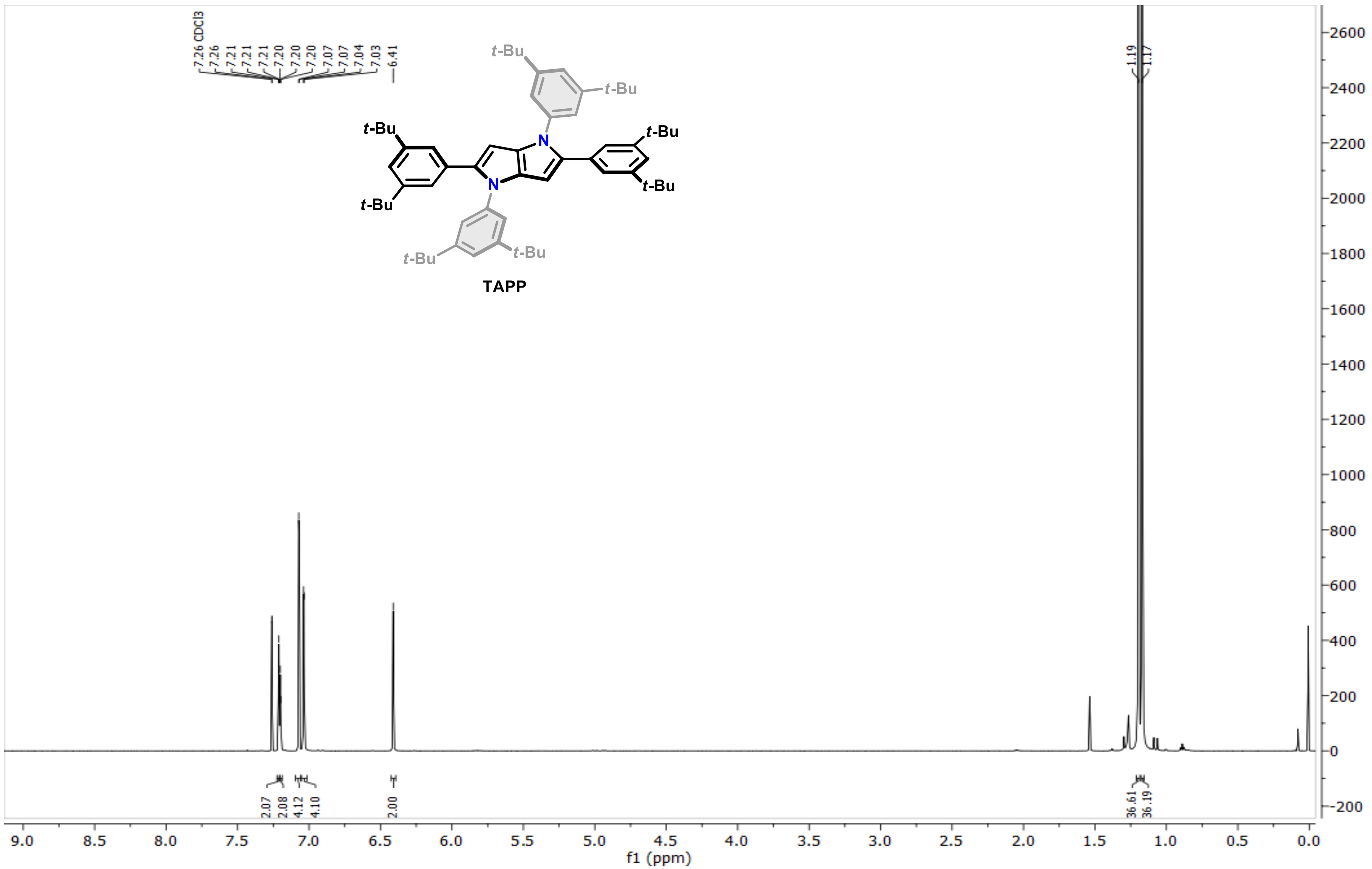

Figure S1. ^{1}H NMR spectrum of **TAPP** (600 MHz, $CDCl_3$).

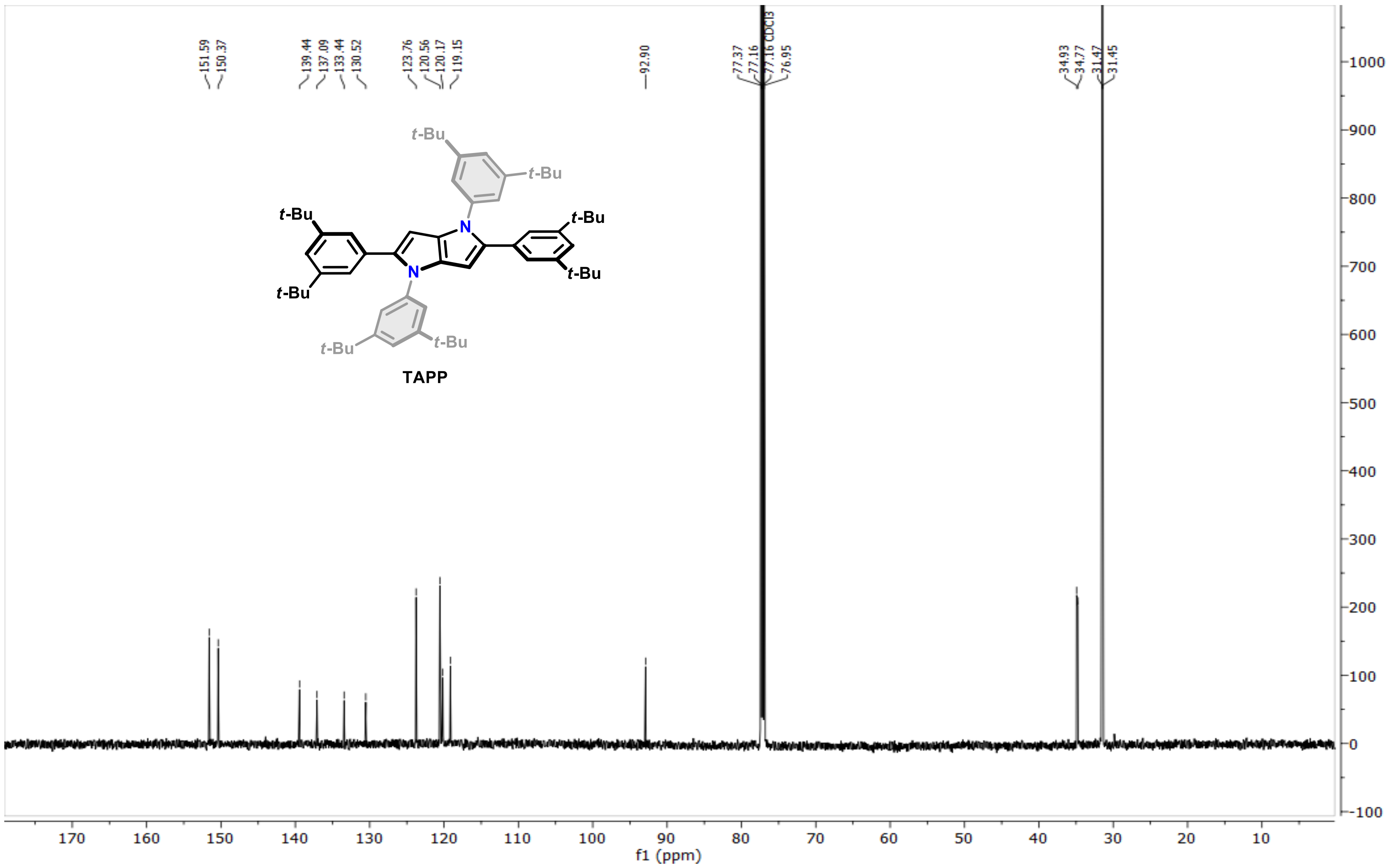

Figure S2. ^{13}C NMR spectrum of **TAPP** (151 MHz, $CDCl_3$).

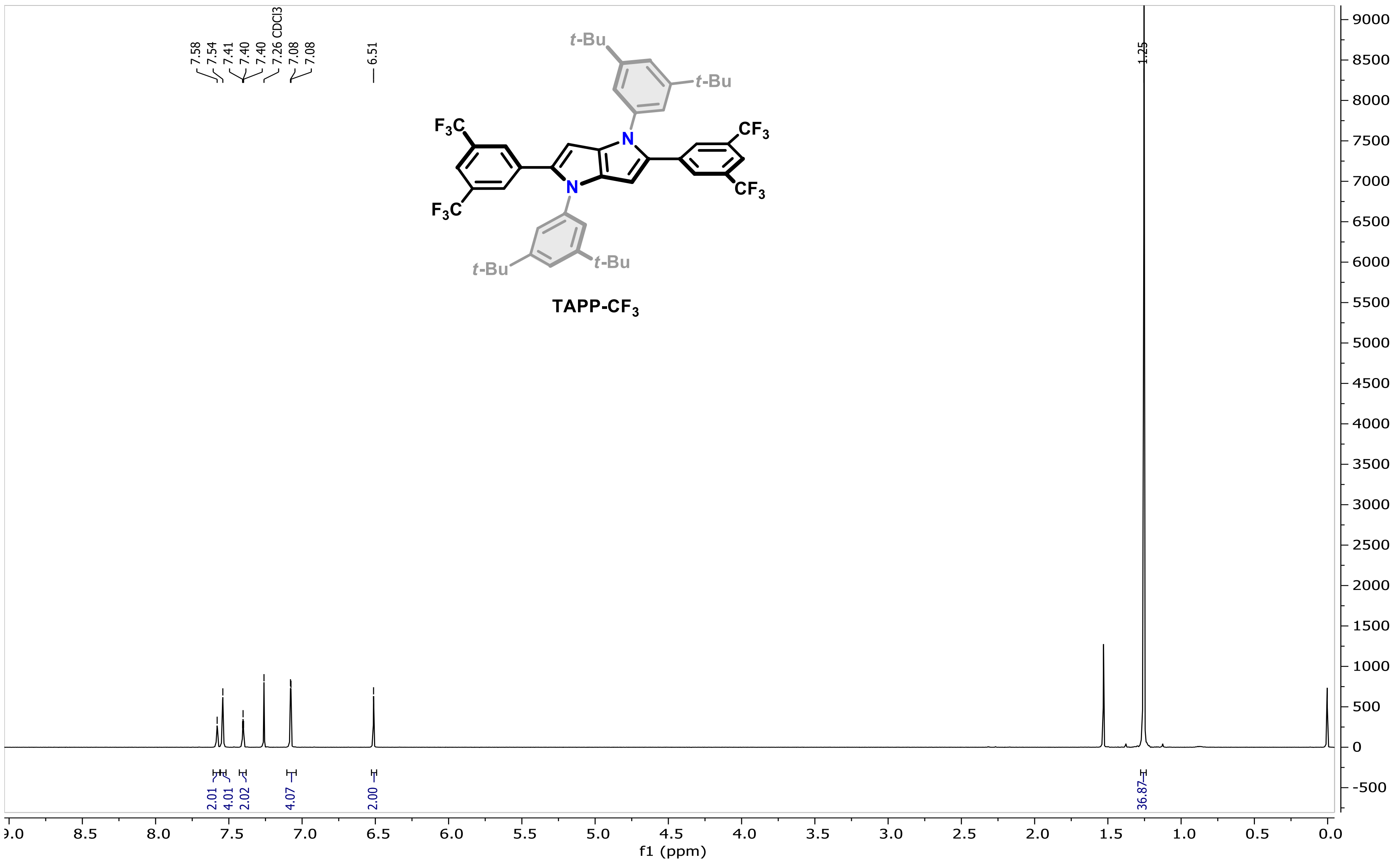

Figure S3. ¹H NMR spectrum of **TAPP-CF$_3$** (500 MHz, CDCl$_3$).

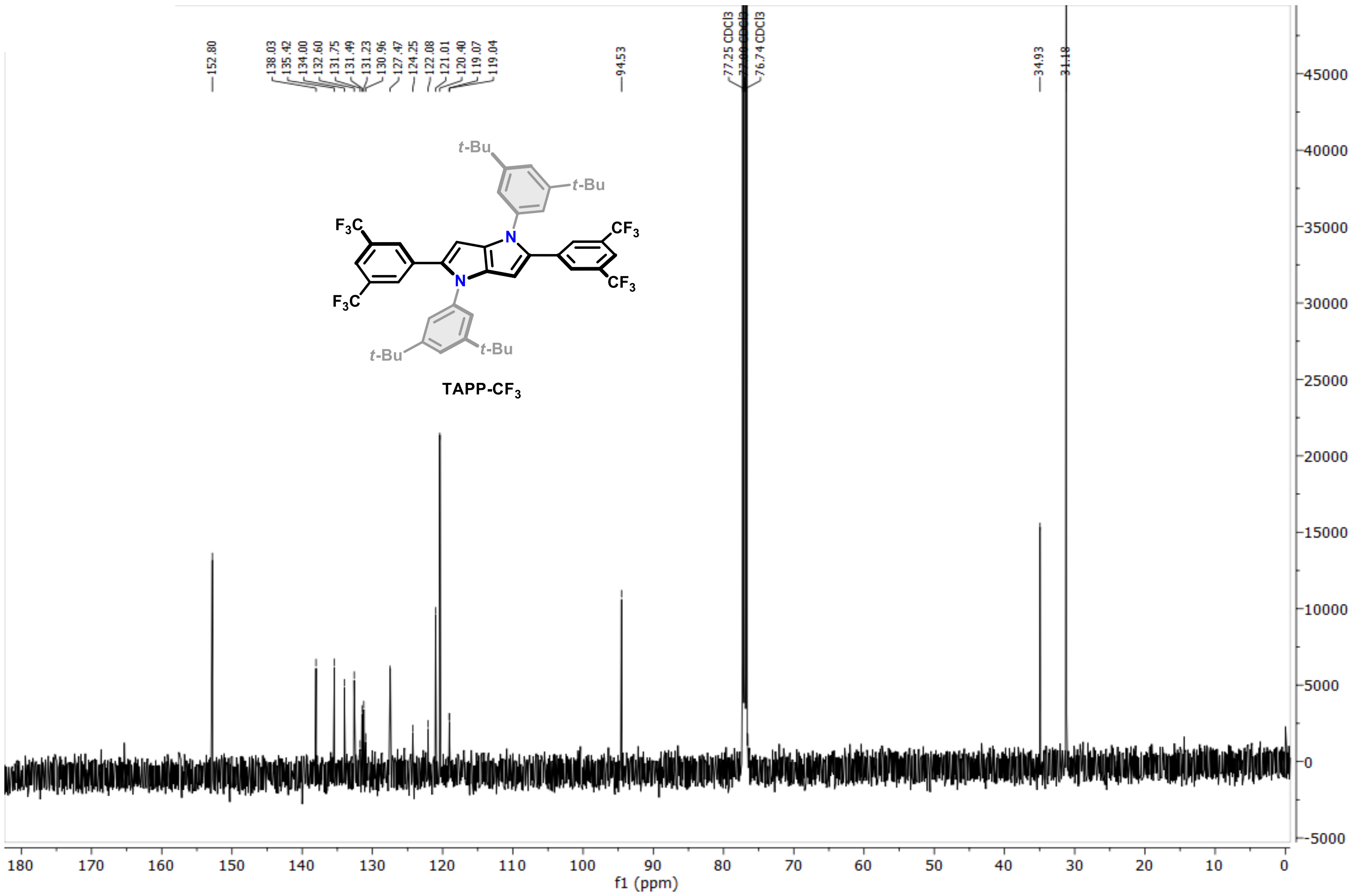

Figure S4. ^{13}C NMR spectrum of **TAPP-CF**$_3$ (126 MHz, $CDCl_3$).

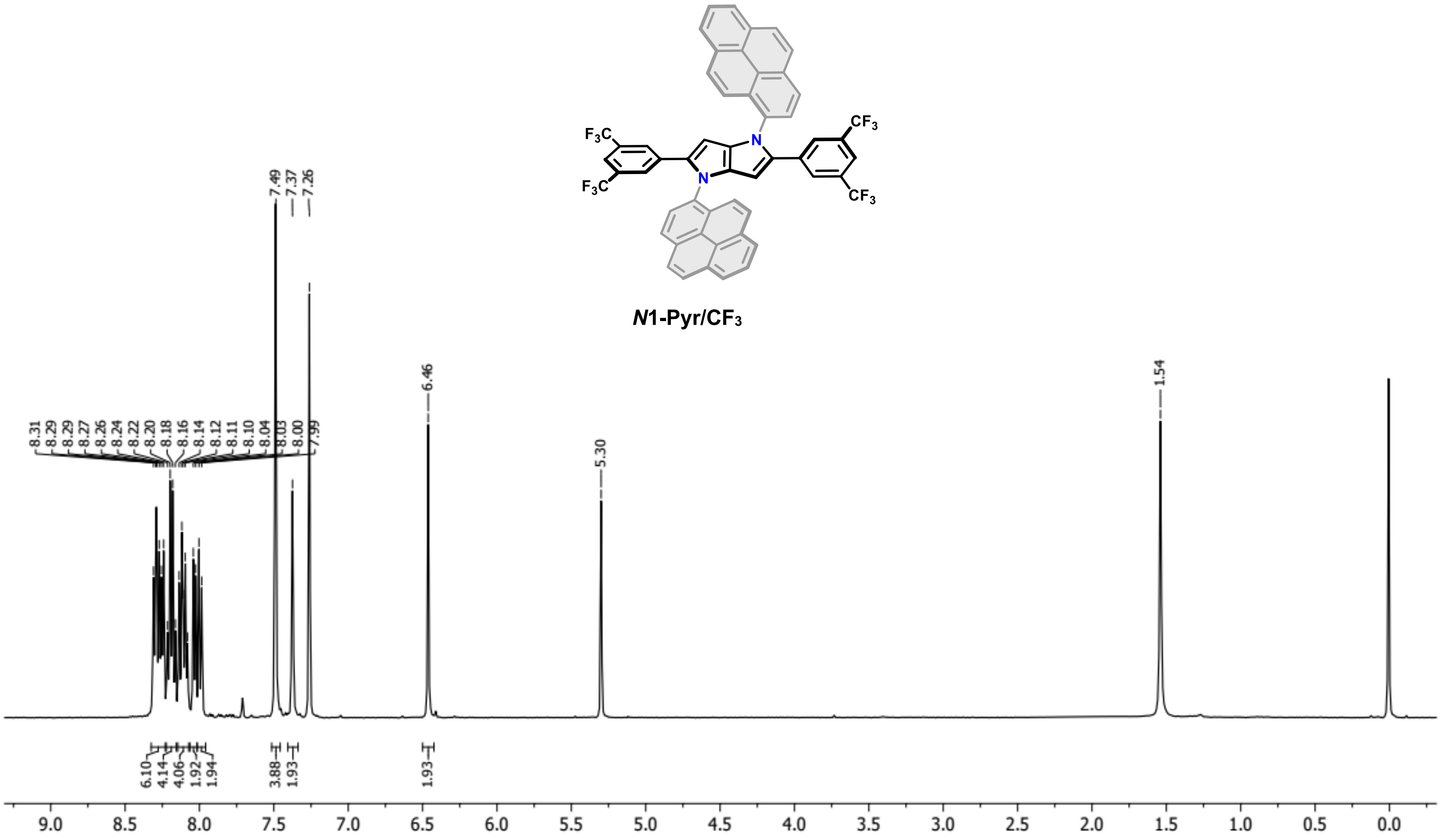

Figure S5. ^{1}H NMR spectrum of ***N*1-Pyr/CF₃** (500 MHz, $CDCl_3$).

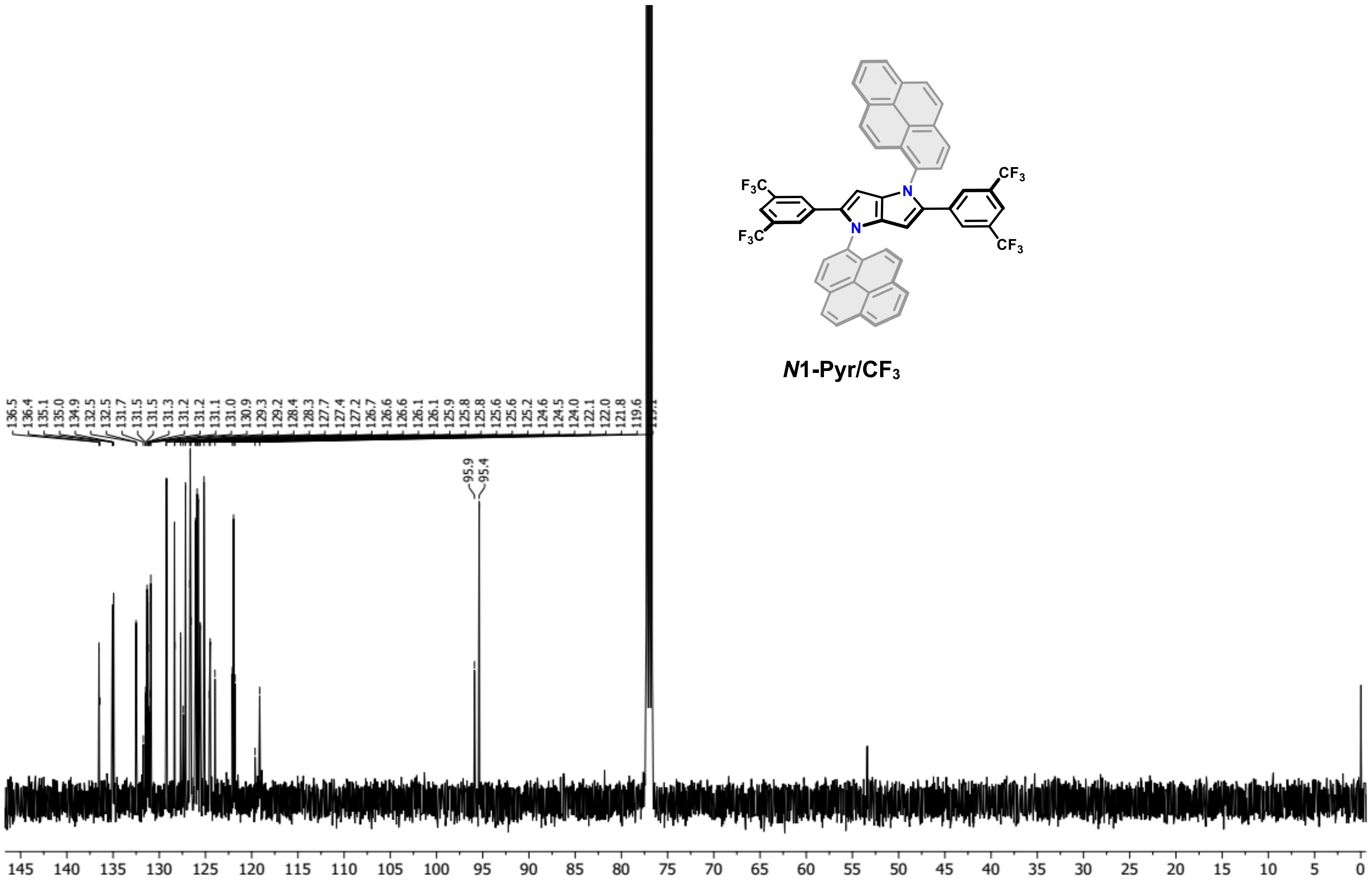

Figure S6. ^{13}C NMR spectrum of ***N*1-Pyr/CF$_3$** (126 MHz, $CDCl_3$).

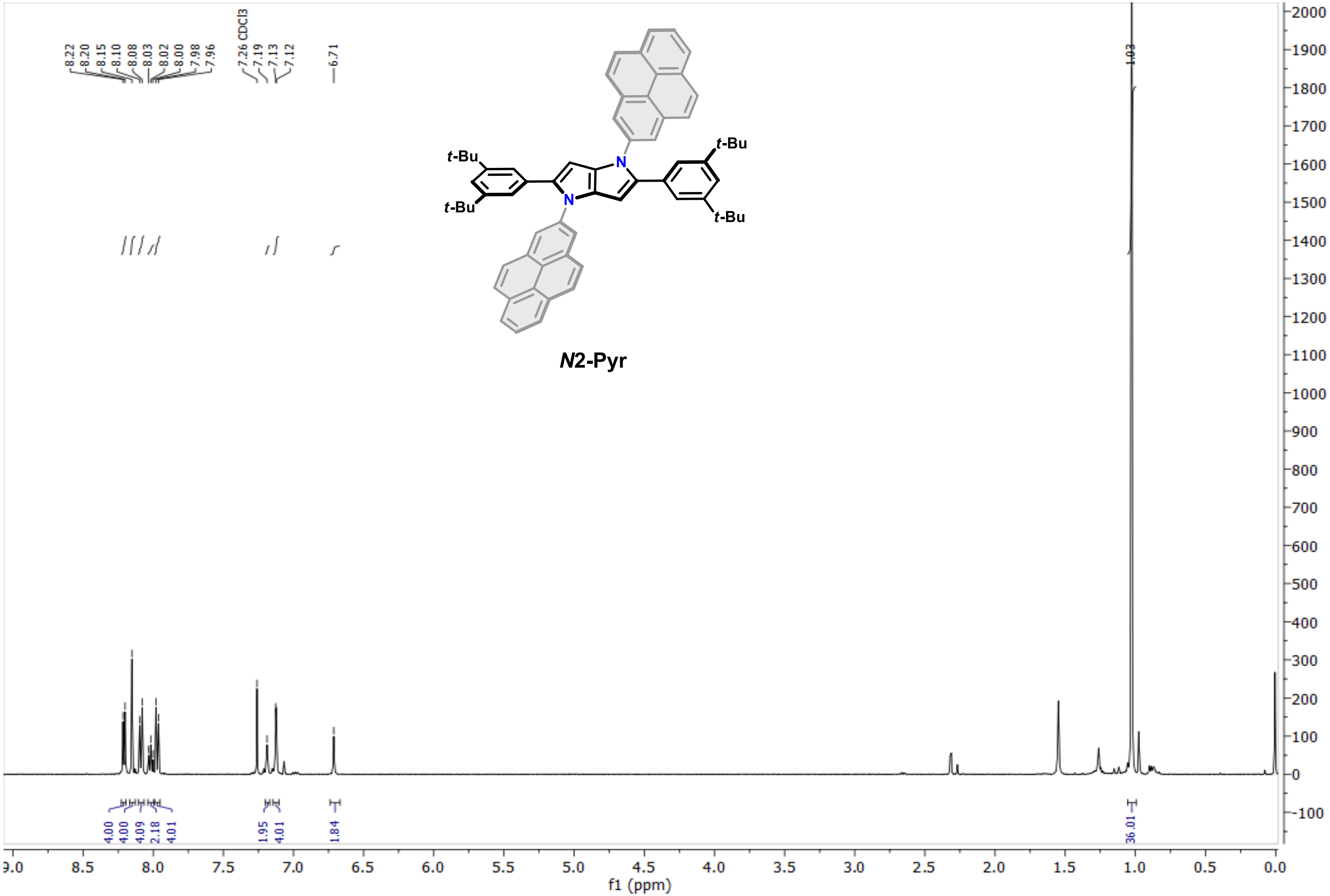

Figure S7. ^{1}H NMR spectrum of ***N*2-Pyr** (500 MHz, $CDCl_3$).

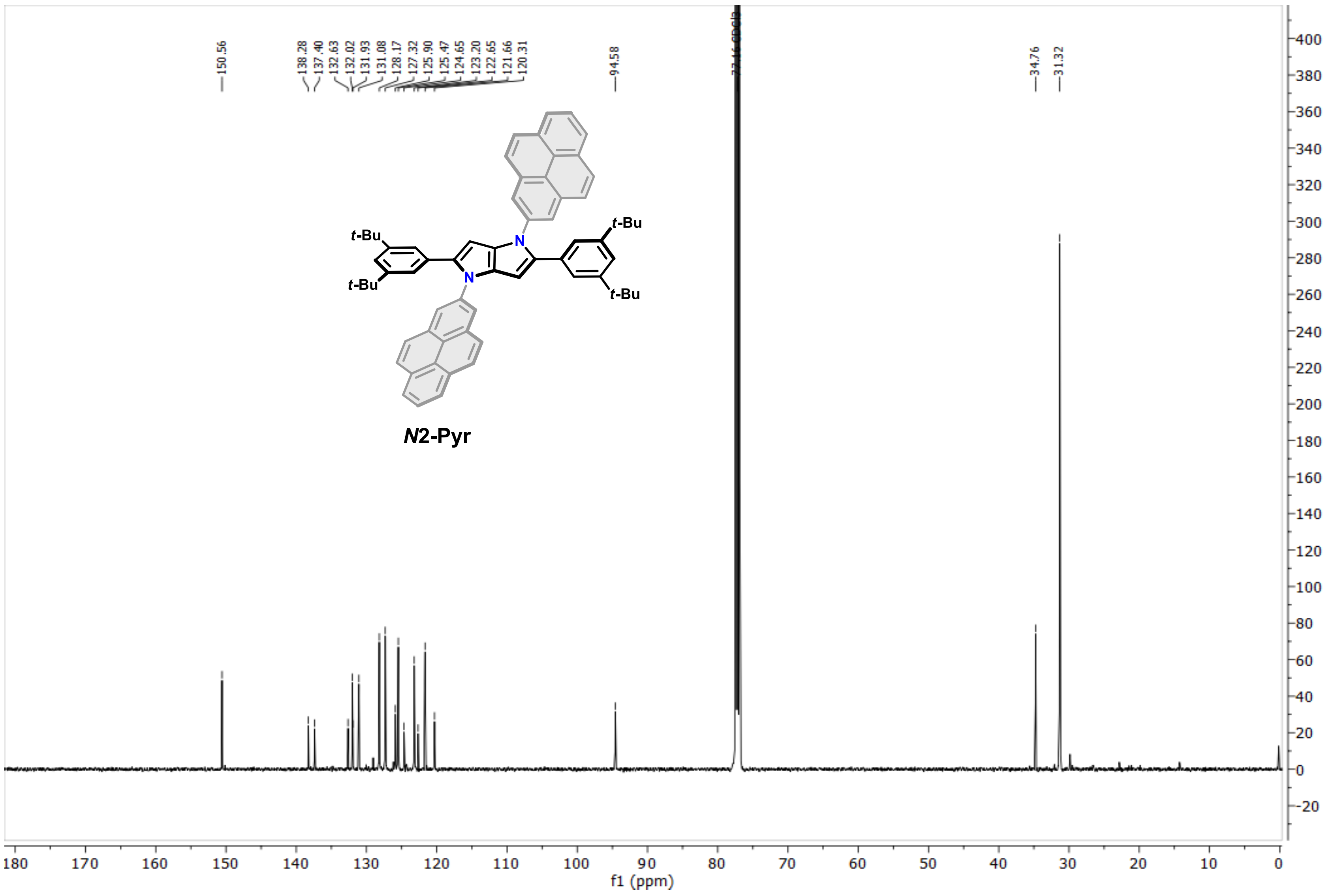

Figure S8. ^{13}C NMR spectrum of ***N*2-Pyr** (126 MHz, $CDCl_3$).

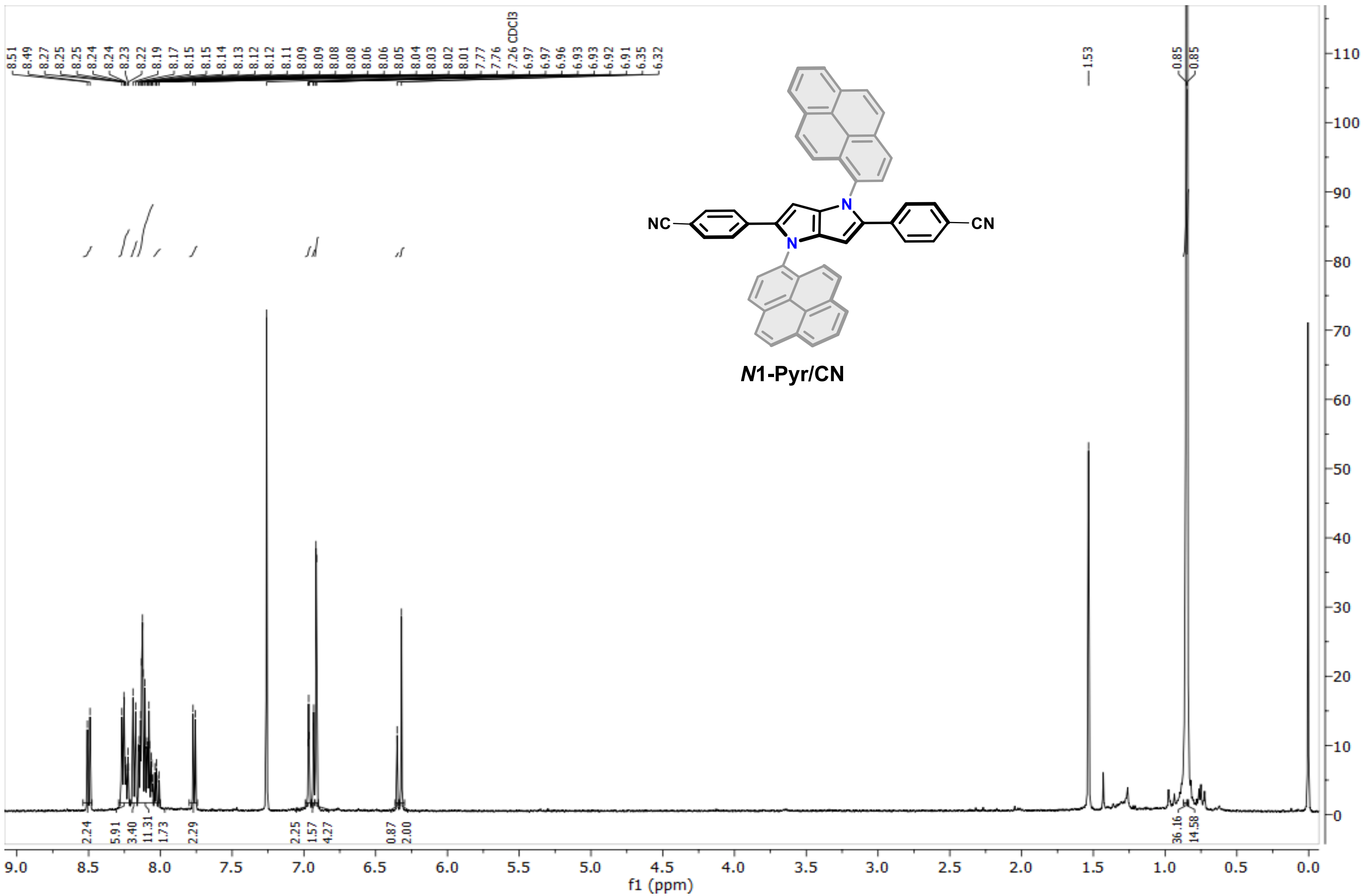

Figure S9. ^{1}H NMR spectrum of ***N*1-Pyr/CN** (500 MHz, DMSO-*d6*).

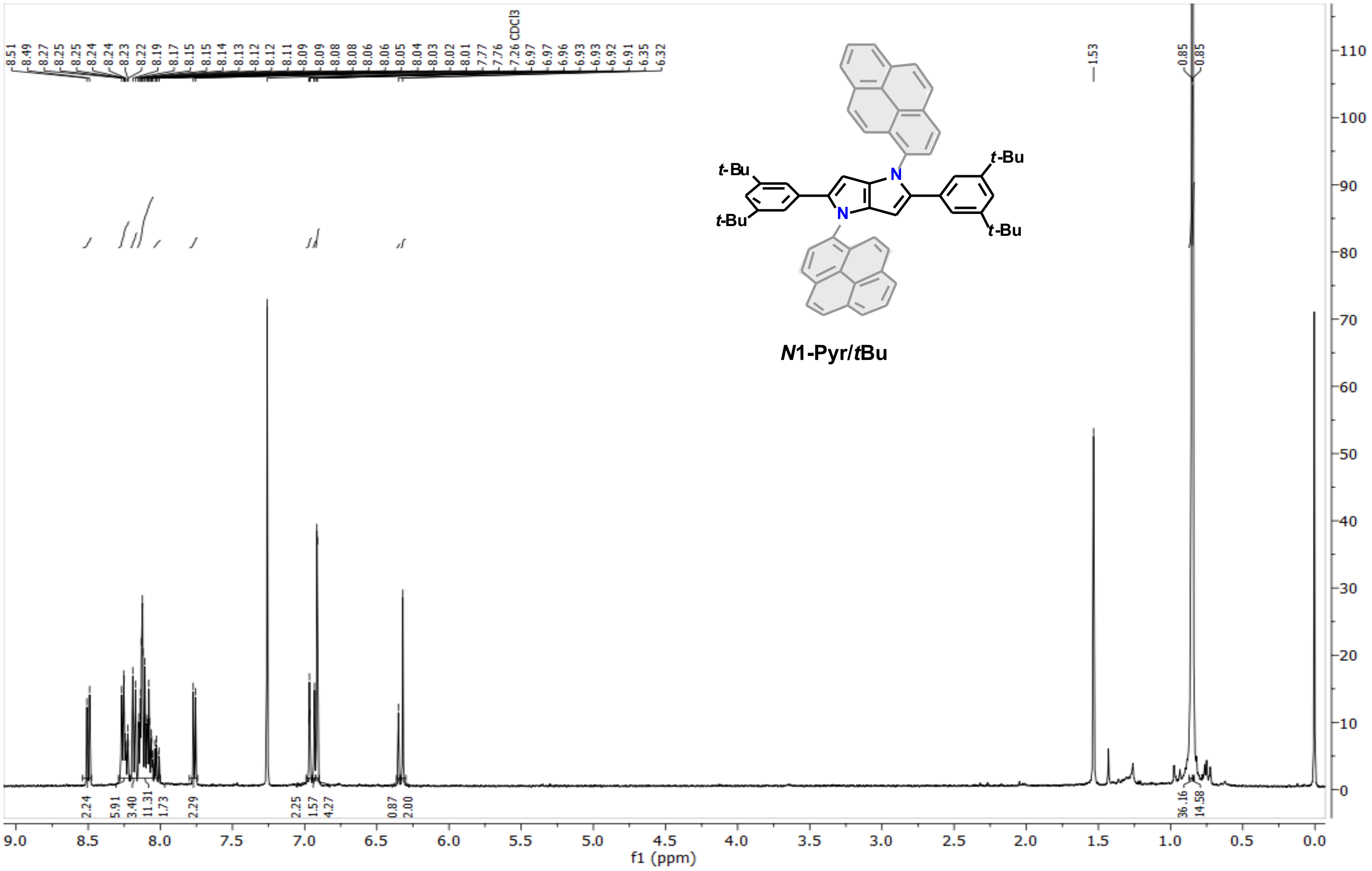

Figure S10. ^{1}H NMR spectrum of ***N*1-Pyr/*t*Bu** (500 MHz, $CDCl_3$).

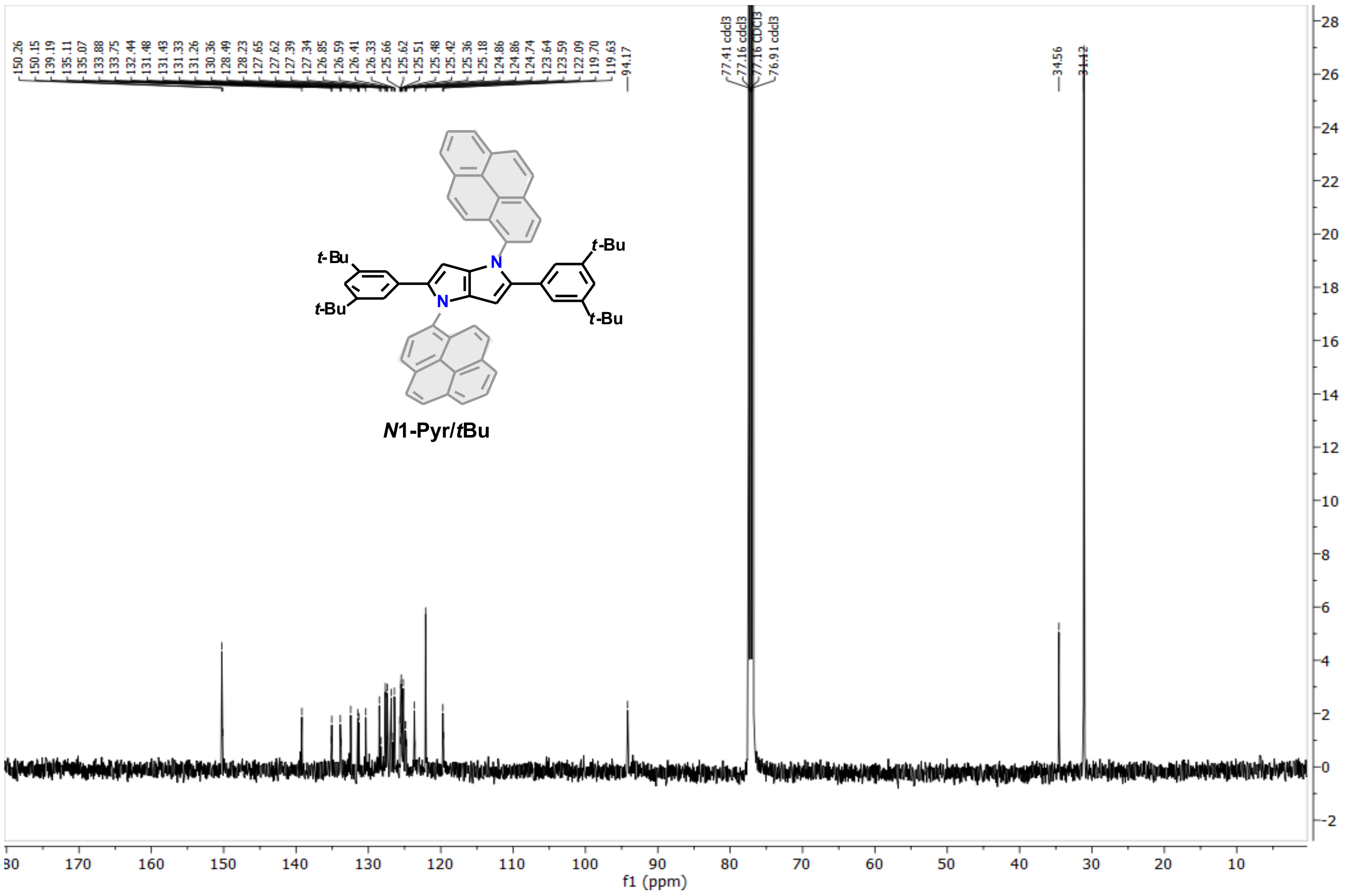

Figure S11. ^{13}C NMR spectrum of ***N*1-Pyr/*t*Bu** (126 MHz, $CDCl_3$).

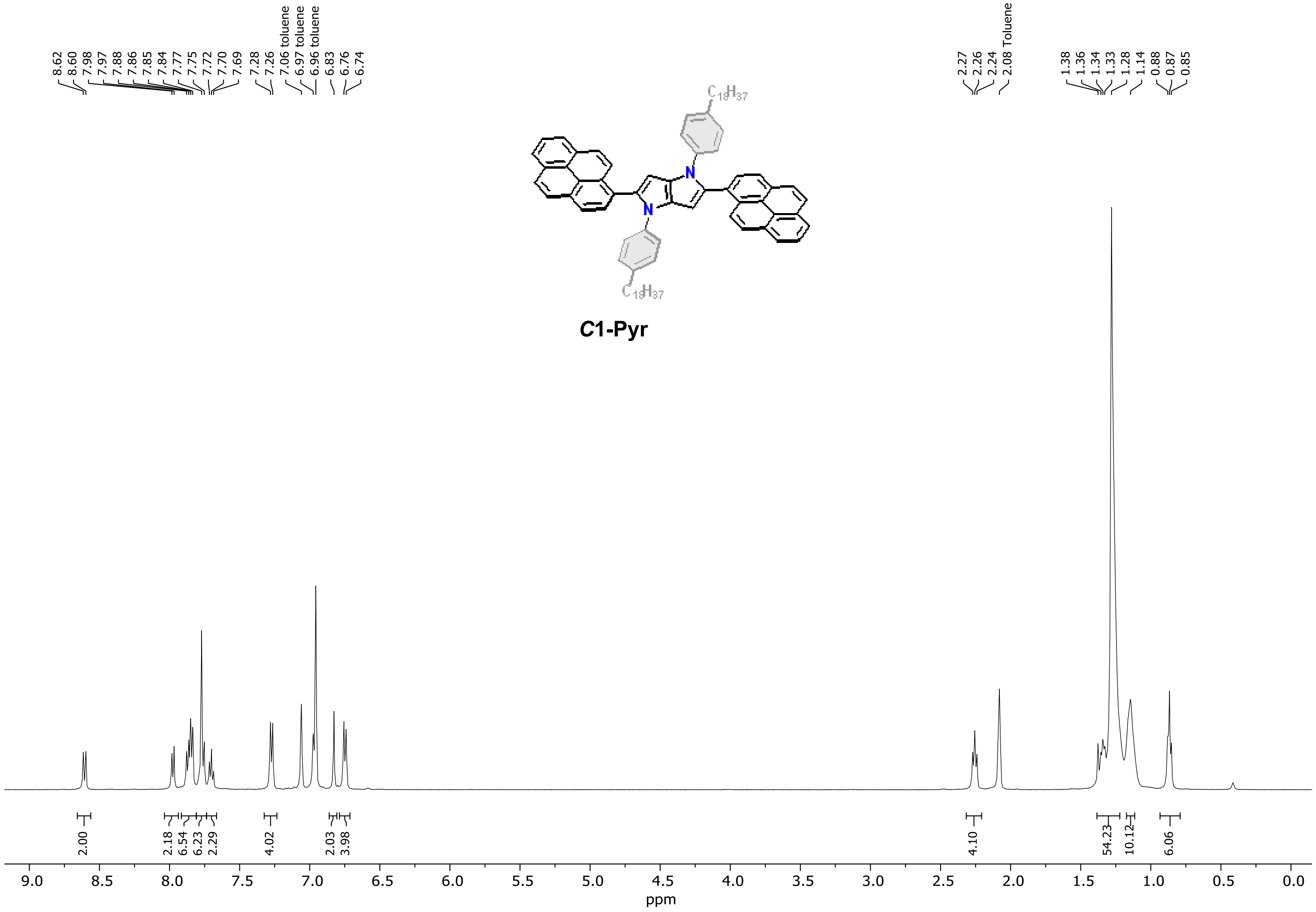

Figure S12. ^{1}H NMR spectrum of ***C*1-Pyr** (500 MHz, toluene-d_8, 80°C).

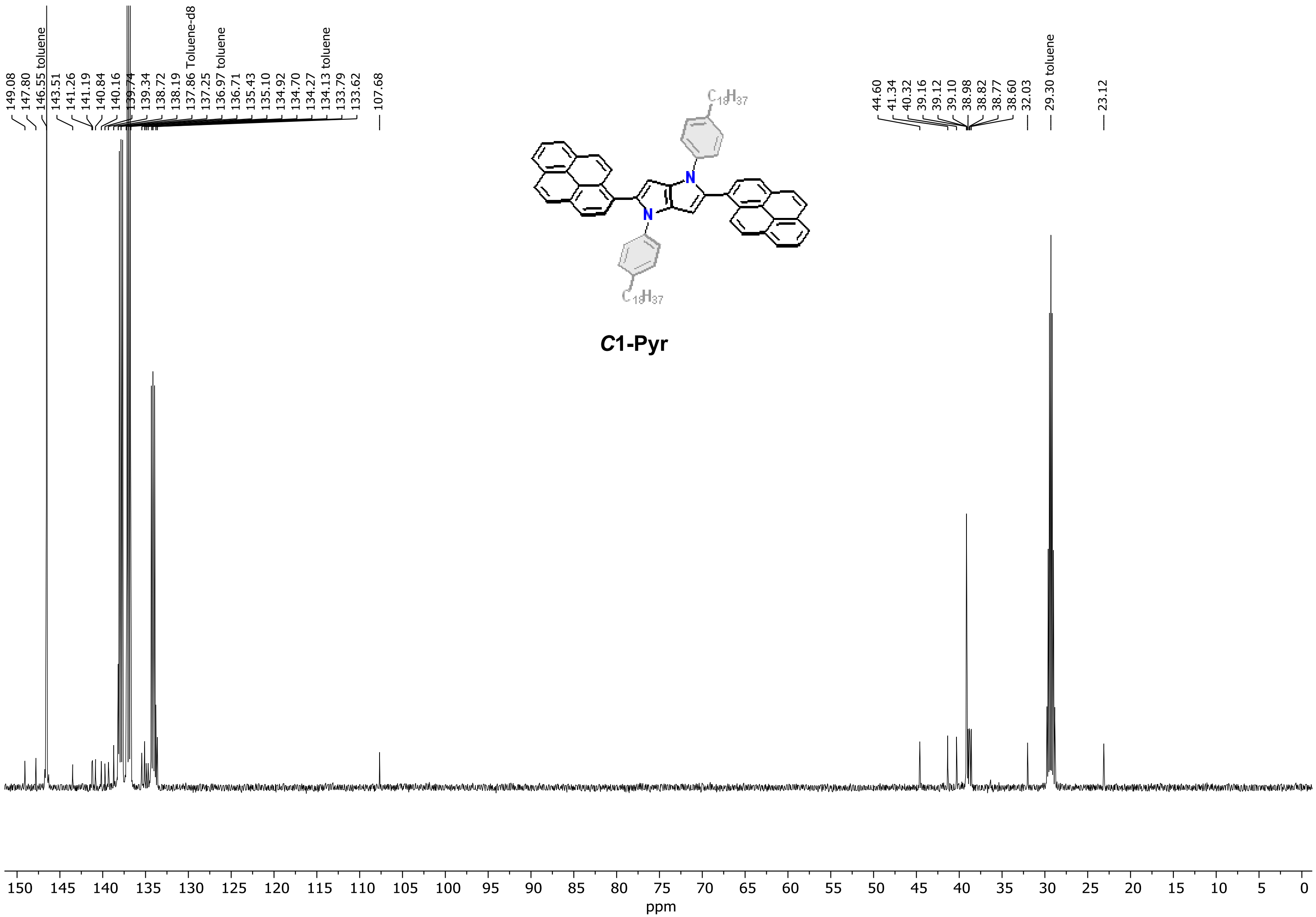

Figure S13. ^{13}C NMR spectrum of ***C*1-Pyr** (126 MHz, toluene-d$_8$, 80°C).

Methods

Steady-state optical spectroscopy and emission decays

Spectrophotometric-grade solvents were used for all optical-spectroscopy studies. Steady-state absorption spectra are recorded in a transmission mode using a JASCO V-670 spectrophotometer (Tokyo, Japan). The steady-state emission spectra and the time-correlated single-photon counting (TCSPC) fluorescence decays are measured, using a FluoroLog-3 spectrofluorometer (Horiba-Jobin-Yvon, Edison, NJ, USA), equipped with a pulsed diode laser (λ = 406 nm, 200 ps pulse width) and a pulsed light emitting diode (λ = 278 nm, 1 ns pulse width).

The fluorescence quantum yields, Φ_f (Table 1), are determined by comparing the integrated emission intensities of the samples with the integrated fluorescence of a reference sample with a known fluorescence quantum yield, Φ_{f0}:

$$\Phi_f = \Phi_{f0} \frac{\int F(\lambda) d\lambda}{\int F(\lambda) d\lambda} \times \frac{1-10^{-A_0(\lambda_{ex})}}{1-10^{-A(\lambda_{ex})}} \times \frac{n^2}{n_0^2} \quad \text{(eq. S1)}$$

Where $F(\lambda)$ is the fluorescence intensity at wavelength λ; $A(\lambda_{ex})$ is the absorbance at the excitation wavelength; n is the refractive index of the media; and the subscript "0" indicates the quantities for the reference sample used. The reference sample was an ethanol solution of coumarin 151, Φ_{f0} = 0.53.

The TCSPC emission decays were recorded at the fluorescence maxima. Signals from the Rayleigh scattering of the excitation pulse from MQ water was used for the instrument-response function (IRF). The emission decays were fitted to exponential functions using deconvolution algorithm employing the IRF signals. Table 1 reports the lifetimes from single-exponential fits and the intensity-average lifetimes from multi-exponential fits.

Transient-absorption spectroscopy

Spectrophotometric-grade solvents were used for all transient-absorption (TA) measurements. The TA spectra at each time point, $\Delta A(\lambda, t)$, are recorded in a transmission mode with 2-mm quartz cuvettes using Helios pump-probe spectrometer (Ultrafast systems, LLC, Florida, USA) equipped with a 3.2-ns delay stage allowing 7-fs temporal step resolution. The laser source for the Helios is a SpitFire Pro 35F regenerative amplifier (Spectra Physics, Newport, CA, USA) generating 800-nm pulses (≥35 fs, 4.0 mJ, at 1 kHz). The SpitFire amplifier is pumped with of an Empower 30 Q-switched laser ran at 20 W at the 2nd harmonic; and a MaiTai SP oscillator provides the seed beam (55 nm bandwidth). 2nd-harmonic and 3rd-harmonic generators of the 800-nm pulses provided 400-nm and 266-nm pump, respectively; and attenuated 800-nm pulses, passed through a Ti:Sapph white-light generator, provided the probe. Global-fit (GF) analysis of the TA data, $\Delta A(\lambda, t)$, yields the evolution associated difference spectra (EADS) with the corresponding time constants, τ, of the sequential transitions.

Spectroscopy of TAPP:

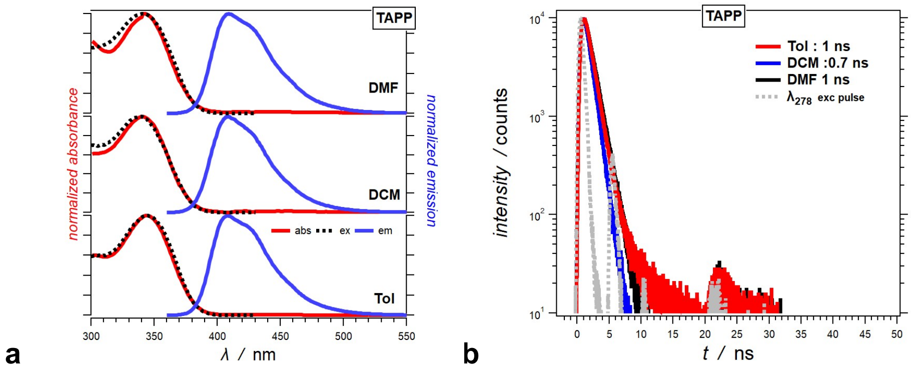

Figure S14. (a) Steady state absorption (abs), emission (em) and excitation(ex) spectra of **TAPP** for toluene (Tol), dichloromethane (DCM) and *N,N*-dimethylformamide (DMF). For the emission spectra, λ_{ex} = 350 nm, and for excitation spectra, λ_{em} = 440 nm. (b) TCSPC emission decays of **TAPP** for Tol, DCM, and DMF (λ_{ex} = 278 nm; λ_{em} = 400 nm) along with the corresponding lifetimes obtained from monoexponentially fits.

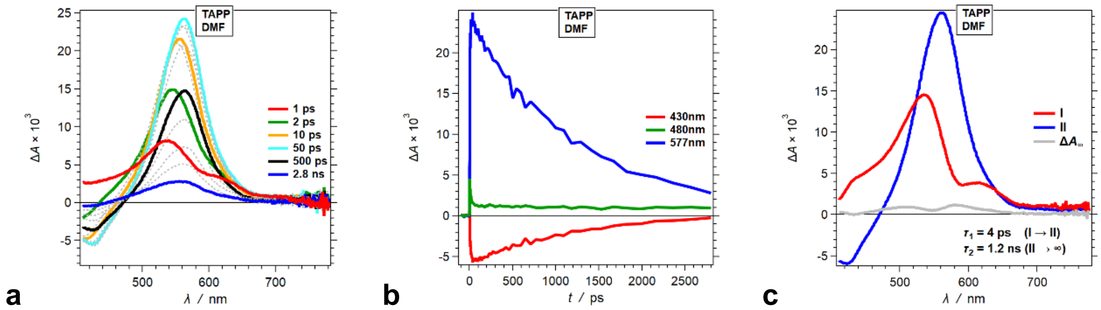

Figure S15. Transient absorption dynamics of **TAPP** for DMF (λ_{ex} = 266 nm). (a) Representative TA spectra. (b) TA kinetic curves depicting rises and decays at selected wavelengths. (c) Evolution associated difference spectra (EADS) with the corresponding transition times constants, τ, obtained from global-fit (GF) analysis of the TA data.

Spectroscopy of TAPP-CF$_3$:

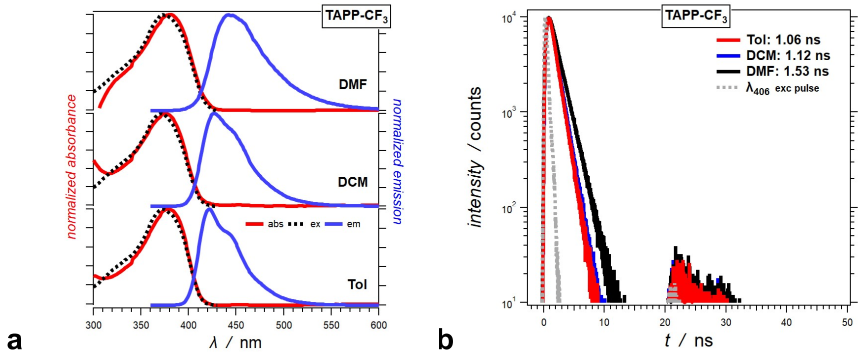

Figure S16. (a) Steady state absorption (abs), emission (em) and excitation(ex) spectra of **TAPP-CF₃** for toluene (Tol), dichloromethane (DCM) and *N,N*-dimethylformamide (DMF). For the emission spectra, λ_{ex} = 350 nm, and for excitation spectra, λ_{em} = 440 nm. (b) TCSPC emission decays of **TAPP-CF₃** for Tol, DCM, and DMF (λ_{ex} = 406 nm; λ_{em} = 440 nm) along with the corresponding lifetimes obtained from monoexponentially fits.

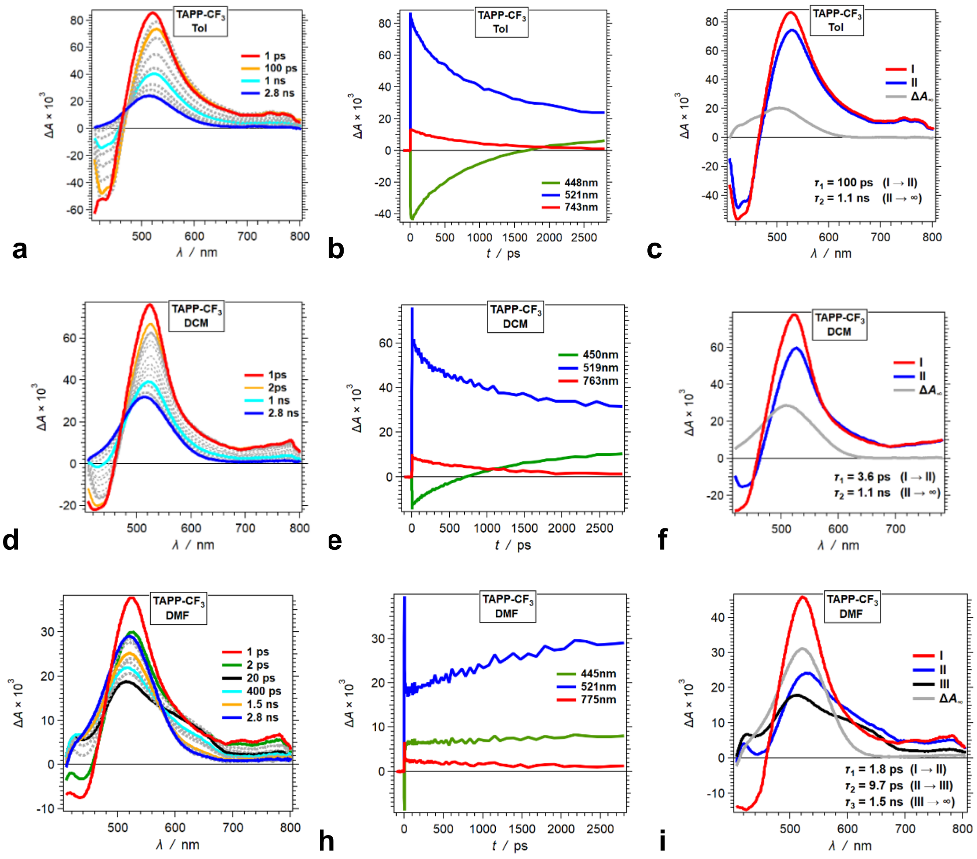

Figure S17. Transient absorption dynamics of **TAPP-CF₃** for (a-c) DMF, (d-f) DCM, and (g-i) DMF (λ_{ex} = 400 nm). (a,d,g) Representative TA spectra. (b,e,h) TA kinetic curves depicting rises and decays at selected wavelengths. (c,f,i) Evolution associated difference spectra (EADS) with the corresponding transition times constants, τ, obtained from global-fit (GF) analysis of the TA data. (d) and (f) are figures 5e and 5f, respectively, shown here for visualizing the trends of solvent-polarity effects.

Spectroscopy of *C*1-Pyr:

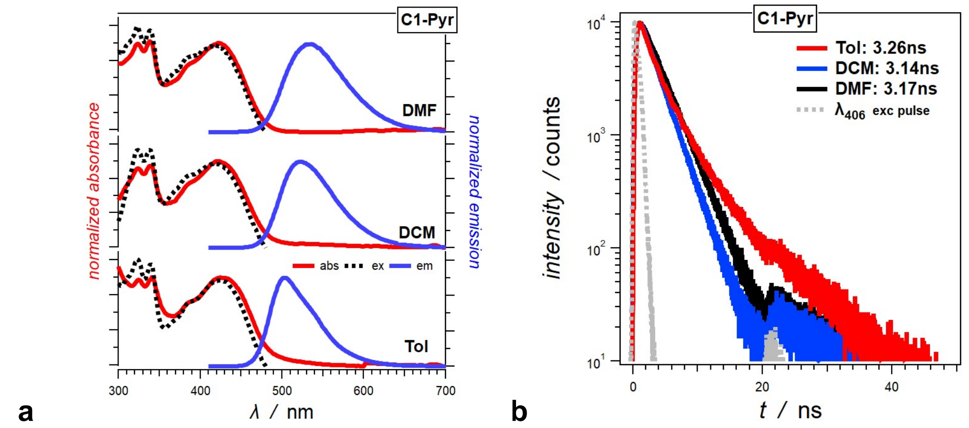

Figure S18. (a) Steady state absorption (abs), emission (em) and excitation(ex) spectra of ***C*1-Pyr** for toluene (Tol), dichloromethane (DCM) and *N,N*-dimethylformamide (DMF). For the emission spectra, λ_{ex} = 400 nm, and for excitation spectra, λ_{em} = 500 nm. (b) TCSPC emission decays of ***C*1-Pyr** for Tol, DCM, and DMF (λ_{ex} = 406 nm; λ_{em} = 500 nm) along with the corresponding lifetimes obtained from monoexponentially fits for DCM and DMF, and intensity average lifetime from biexponential for toluene.

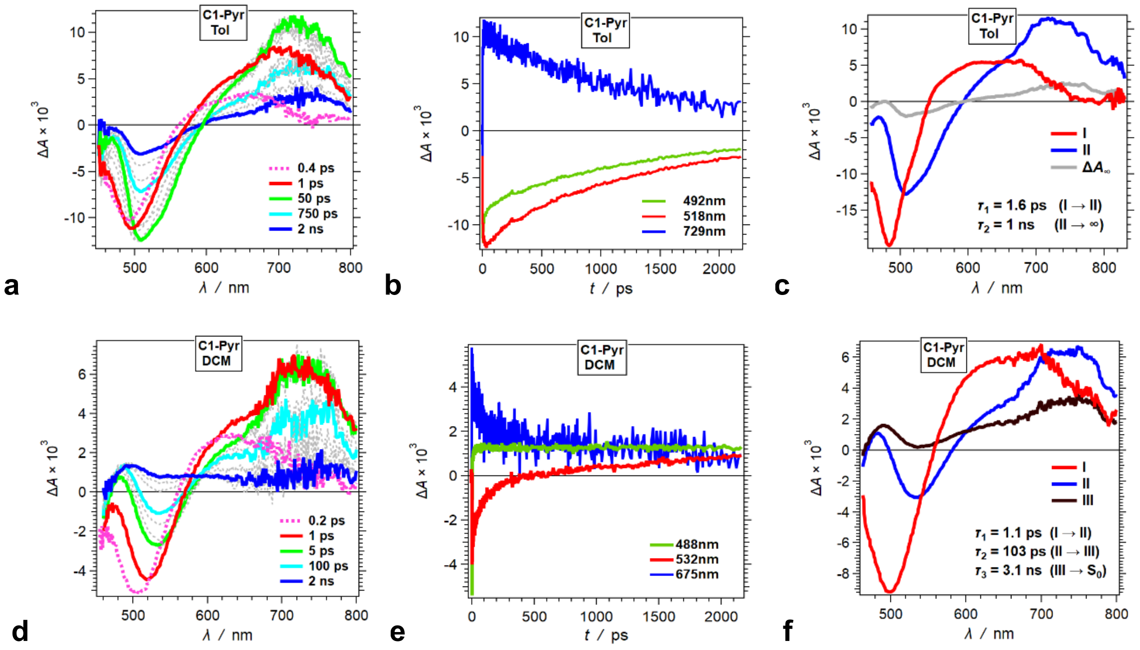

Figure S19. Transient absorption dynamics of ***C*1-Pyr** for (a-c) DMF and (d-f) DCM (λ_{ex} = 400 nm). (a,d) Representative TA spectra. (b,e) TA kinetic curves depicting rises and decays at selected wavelengths. (c,f) Evolution associated difference spectra (EADS) with the corresponding transition times constants, τ, obtained from global-fit (GF) analysis of the TA data.

Spectroscopy of *N*1-Pyr/*t*Bu:

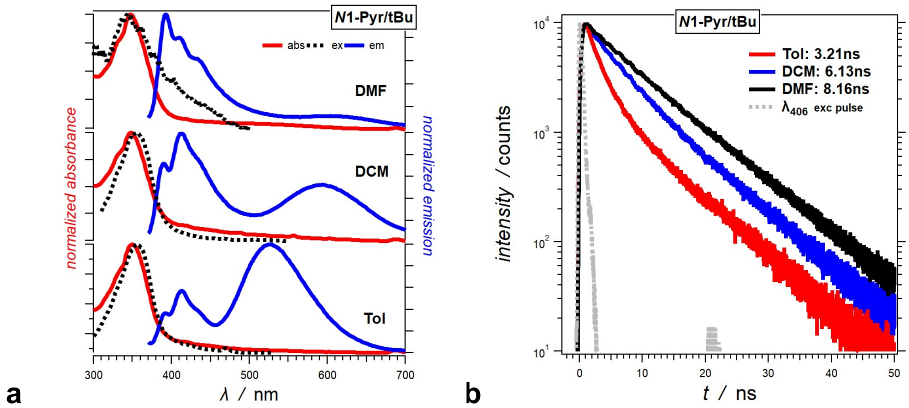

Figure S20. (a) Steady state absorption (abs), emission (em) and excitation(ex) spectra of ***N*1-Pyr/*t*Bu** for toluene, DCM and DMF. For the emission spectra, λ_{ex} = 350 nm, and for excitation spectra, λ_{em} = 540 nm for toluene, 560 nm for DCM and DMF. (b) TCSPC emission decays of ***N*1-Pyr/*t*Bu** for toluene, DCM, and DMF (λ_{ex} = 406 nm; λ_{em} = 540 nm for toluene, 560 nm for DCM and DMF) along with the corresponding lifetimes obtained from monoexponentially fits for toluene and DCM.

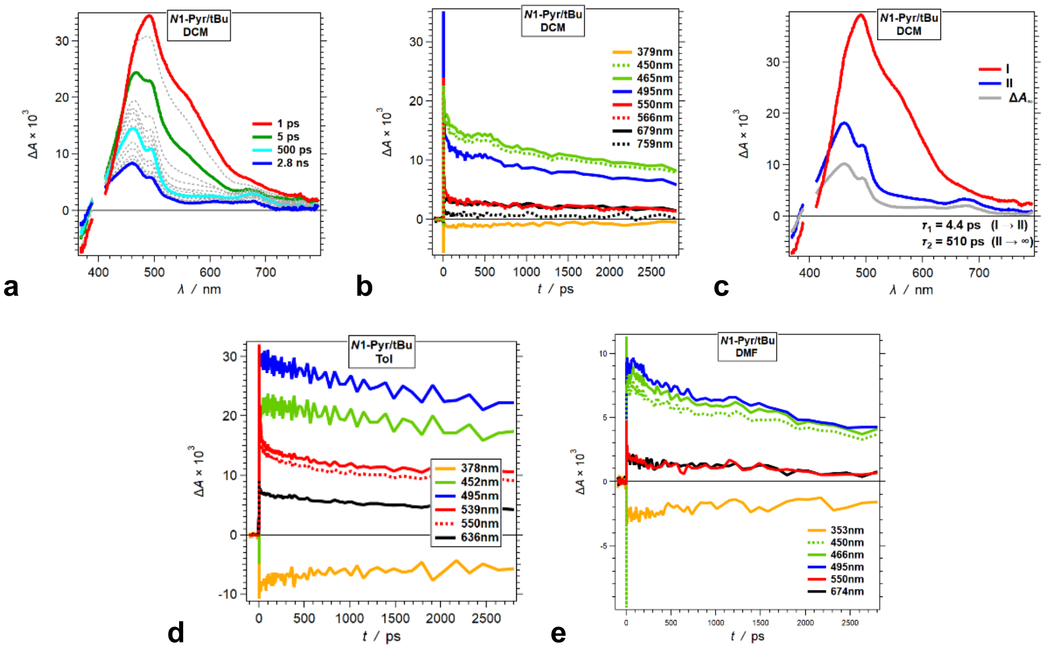

Figure S21. Transient absorption absorption dynamics of ***N*1-Pyr/*t*Bu** for (a-c) DCM, (d) toluene and (e) DMF (λ_{ex} = 400 nm). (a) Representative TA spectra. (b,d,e) TA kinetic curves depicting rises and decays at selected wavelengths. (c) Evolution associated difference spectra (EADS) with the corresponding transition times constants, τ, obtained from global-fit (GF) analysis of the TA data.

Spectroscopy of *N*2-Pyr:

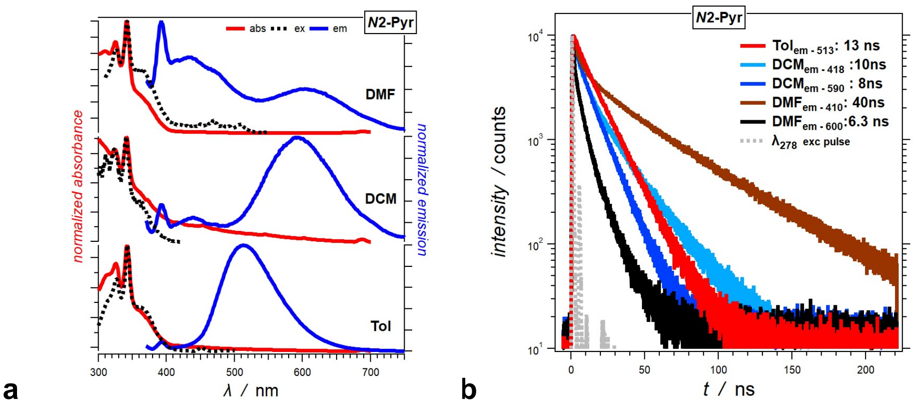

Figure S22. (a) Steady state absorption (abs), emission (em) and excitation(ex) spectra of ***N*2-Pyr** for toluene, DCM and DMF. For the emission spectra, λ_{ex} = 350 nm, and for excitation spectra, λ_{em} = 510 nm for toluene, 600 nm for DCM and DMF. (b) TCSPC emission decays of ***N*2-Pyr** for toluene, DCM, and DMF recorded at different emission wavelengths (λ_{ex} = 406 nm) along with the corresponding lifetimes.

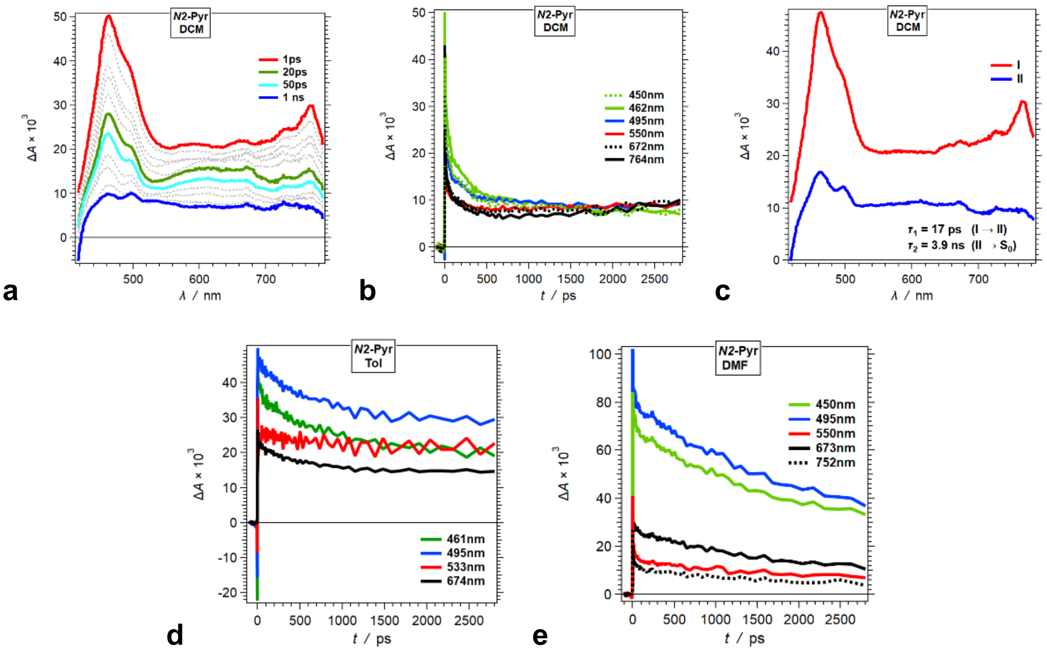

Figure S23. Transient absorption dynamics of ***N*2-Pyr** for (a-c) DCM, (d) toluene and © DMF (λ_{ex} = 400 nm). (a) Representative TA spectra. (b,d,e) TA kinetic curves depicting rises and decays at selected wavelengths. (c) Evolution associated difference spectra (EADS) with the corresponding transition times constants, τ, obtained from global-fit (GF) analysis of the TA data.

Spectroscopy of *N*1-Pyr/CF₃:

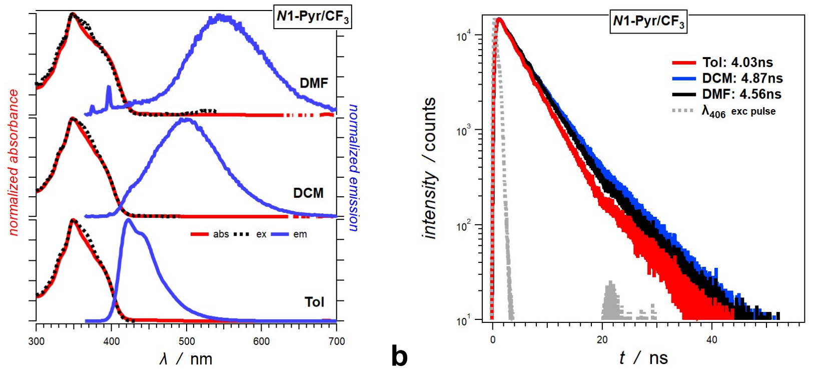

Figure S24. (a) Steady state absorption (abs), emission (em) and excitation(ex) spectra of ***N*1-Pyr/CF₃** for toluene, DCM and DMF. For the emission spectra, λ_{ex} = 350 nm, and for excitation spectra, λ_{em} = 440 nm for toluene, 500 nm for DCM and 550 nm for DMF. (b) TCSPC emission decays of ***N*1-Pyr/CF₃** for toluene, DCM, and DMF (λ_{ex} = 406 nm; λ_{em} = 440 nm for toluene, 500 nm for DCM and 550 nm for DMF) along with the corresponding lifetimes obtained from monoexponentially fits and intensity average lifetime from biexponential for toluene.

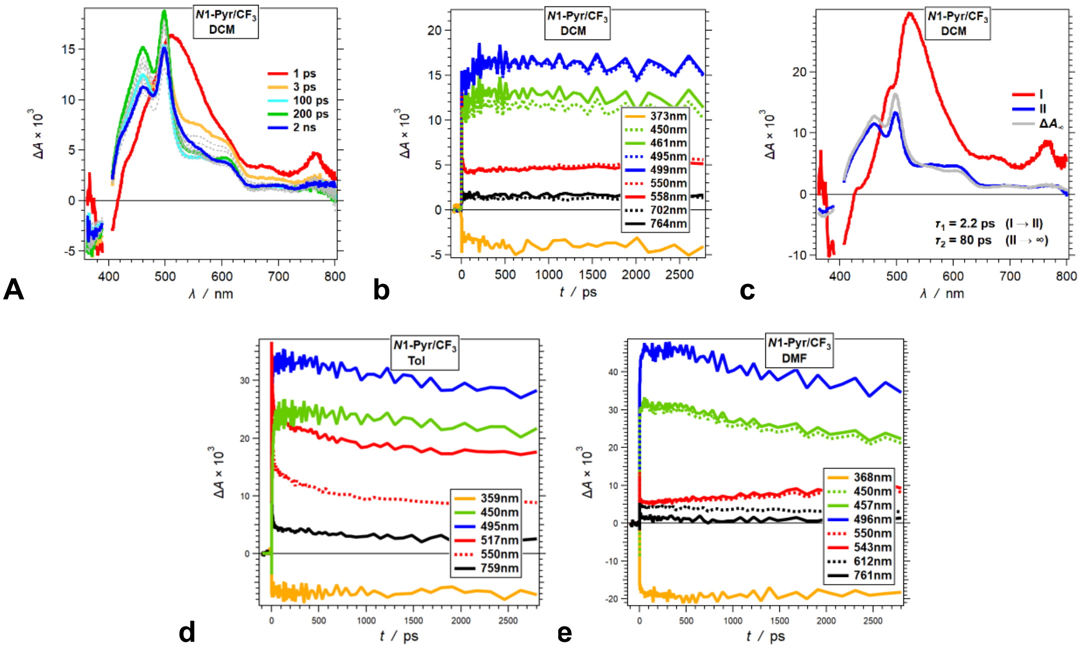

Figure S25. Transient absorption dynamics of ***N*1-Pyr/CF₃** for (a-c) DCM, (d) toluene and (e) DMF (λ_{ex} = 400 nm). (a) Representative TA spectra. (b,d,e) TA kinetic curves depicting rises and decays at selected wavelengths. (c) Evolution associated difference spectra (EADS) with the corresponding transition times constants, τ, obtained from global-fit (GF) analysis of the TA data.

Spectroscopy of *N*1-Pyr/CN:

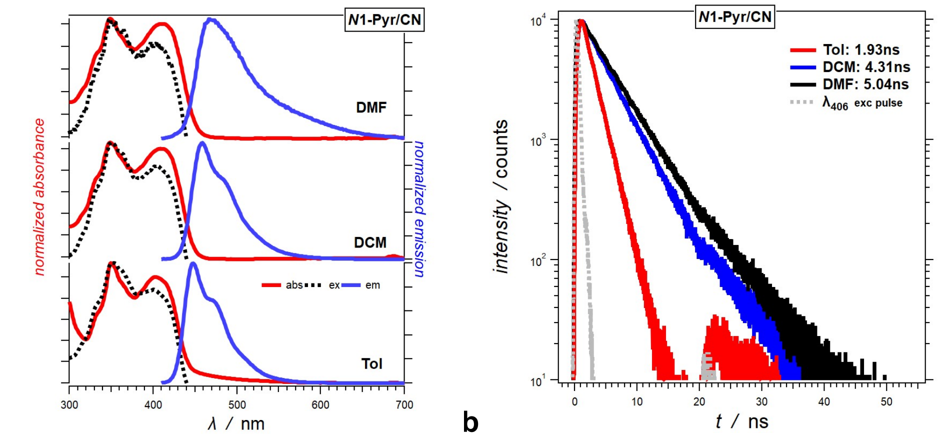

Figure S26. (a) Steady state absorption (abs), emission (em) and excitation(ex) spectra of ***N*1-Pyr/CN** for toluene, DCM and DMF. For the emission spectra, λ_{ex} = 400 nm, and for excitation spectra, λ_{em} = 460 nm. (b) TCSPC emission decays of ***N*1-Pyr/CN** for toluene, DCM, and DMF (λ_{ex} = 406 nm; λ_{em} = 460 nm) along with the corresponding lifetimes obtained from monoexponentially fits.

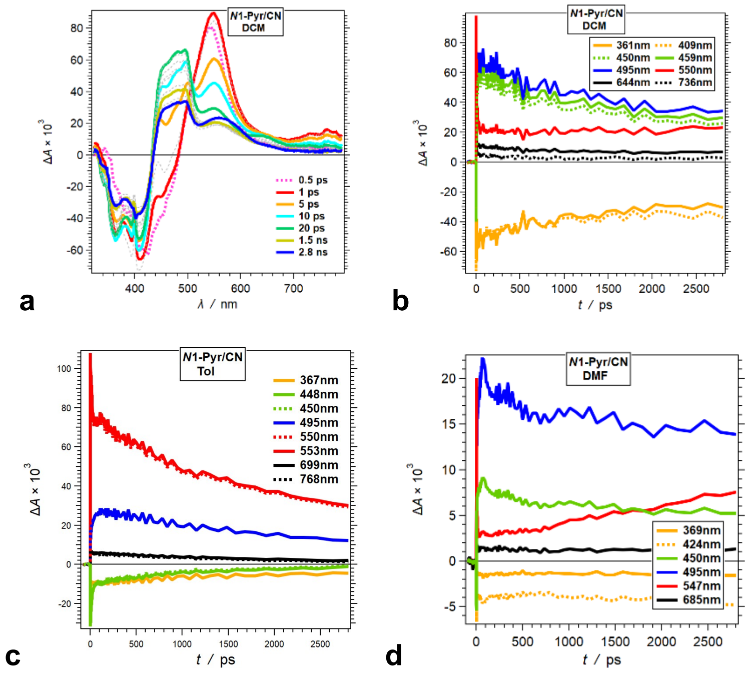

Figure S27. Transient absorption dynamics of ***N*1-Pyr/CN** for (a,b) DCM, (c) toluene and (d) DMF (λ_{ex} = 400 nm). (a) Representative TA spectra. (b-d) TA kinetic curves depicting rises and decays at selected wavelengths.

Dependence of the emission on the excitation wavelength

These N-substituted pyrene-pyrrolopyrroles conjugates tend to manifest fluorescence from different locally excited and CT states. Therefore, we examine the effects of excitation wavelength, λ_{ex}, on the emission spectra of ***N*1-Pyr/tBu** and ***N*2-Pyr** that manifest dual fluorescence, i.e., long-wavelength and short-wavelength emission bands, for all solvents (figure S28 and S29). Shifting λ_{ex} from 310 nm to 400 nm in increments 10, 15, and 20 nm, results in huge variations of the ratio between the amplitudes of the short-wavelength and long-wavelength fluorescence.

Upon excitation at 310 nm, the broad long-wavelength fluorescence exhibits strongest contribution to the emission spectra of ***N*1-Pyr/tBu** and ***N*2-Pyr** for all solvents (Figure S28, S29). It suggests that the upper excited states, accessible at this short wavelength, relax preferentially to an emissive CT state. One cannot preclude also a possibility for a direct transition to an upper CT state, i.e., $S_0 \rightarrow CT^{(FC)}$, with high oscillator strength.

Increasing λ_{ex} to about 340 nm leads to a growth of the relative intensity of the sharp 390-nm peaks, ascribed to fluorescence from the pyrene locally excited state in the spectra of ***N*1-Pyr/tBu** (Figure S28). A further increase in λ_{ex} to 400 mn induces a sharp growth of a mission band ascribed to fluorescence for an excited state localized on the pyrrolopyrrole. For toluene and DCM, when λ_{ex} exceeds 400 mn, only weak long-wavelength emission emerges consistent with direct photoexcitation to a CT state with small osculator strength. For DMF, long excitation wavelengths result in emission spectra dominated by the pyrrolopyrrole fluorescence and lacking the CT band (Figure S28c). Lowering the energy level of the CT state in the polar DMF medium enhances the rates of non-radiative deactivation.

In the case of ***N*1-Pyr**, the largest contributions from the short-wavelength fluorescence emerges as increases from 310 nm to about 340 nm. This wavelength range covers the distinct absorption peaks ascribed to $S_0 \rightarrow S_2$ pyrene transition. Nevertheless, the emission spectra do not reveal such sharp features corresponding to pyrene $S_1 \rightarrow S_0$ radiative deactivation (Figure S29). The short-wavelength emission of ***N*2-Pyr**, however, exhibits an unusually long 40-ns decay components (Figure S22b). Such long-lived emissive states are consistent with the forbidden nature of the pyrene $S_1 \rightarrow S_0$ transition. The pronouncedly weak emission of ***N*2-Pyr** in DMF (regardless λ_{ex}) is consistent with increasing the population with low-lying CT states. It increases not only the rates of ICT, but also the rates of non-radiative deactivation from such low lying CT states.

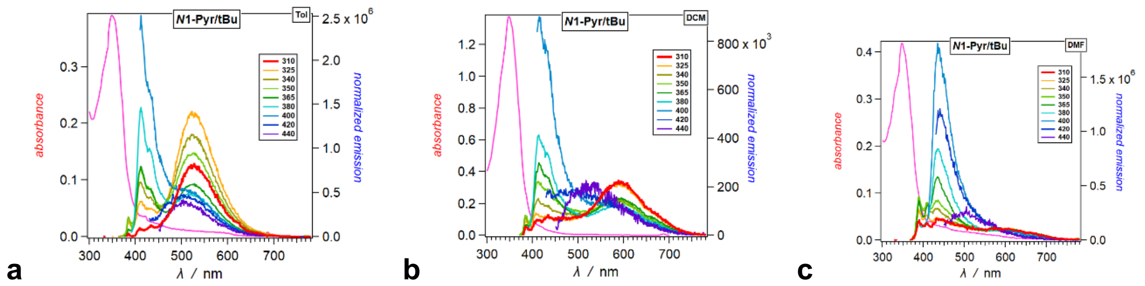

Figure S28. Emission spectra of *N*1-Pyr/*t*Bu excited at various wavelengths from 310nm to 440nm, for (a) toluene, (b) DCM and (c) DMF.

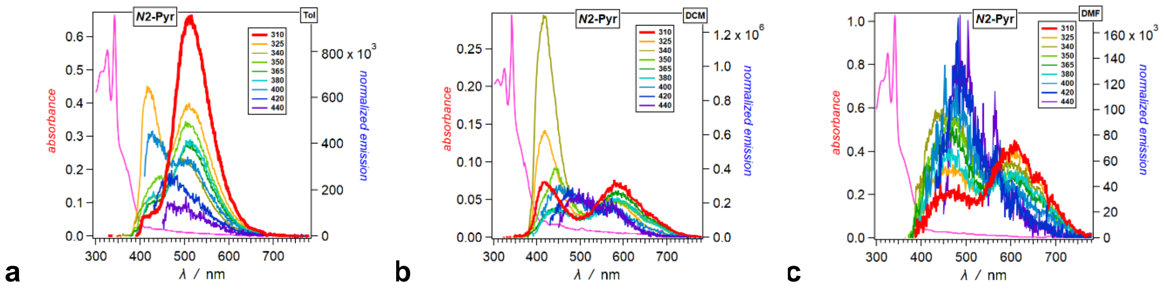

Figure S29. Emission spectra of ***N*2-Pyr** excited at various wavelengths from 310 nm to 440 nm for (a) toluene, (b) DCM and (c) DMF.

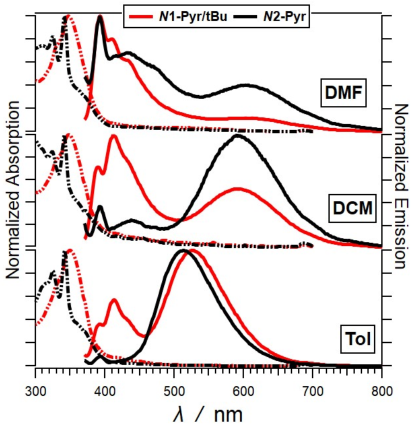

Figure S30. Absorption and emission spectra of ***N*1-Pyr/*t*Bu** and ***N*2-Pyr** in toluene, DCM and DMF, plotted together for comparison (λ_{ex} = 360 nm).

Electrochemistry and spectroelectrochemistry

Anhydrous aprotic solvents were used for all electrochemical measurements and tetrabutylammonium hexafluorophosphate (electrochemistry grade, ≥99.0%) served as a supporting electrolyte. Cyclic voltammetry is conducted using Reference 600^{TM} Potentiostat/Galvanostat/ZRA (Gamry Instruments, PA, U.S.A.), connected to a three-electrode cell. Using the conditions that provide optimal reversibility of the oxidation of the pyrolopyrrole samples, we assembled a three-electrode cell in a 2 mm x 10 mm quartz cuvette placed in a UV/Vis spectrometer. The light was transmited through a platinum mesh that served as a working electrode. The spectra of the intensity of the transmitted light, $I(\lambda, t)$, were recorded at 100-mV increments through the potential sweeps. The differential absorption spectra, $\Delta A(\lambda, t)$, at different time points were obtained by using the initially recorded intensity (at time 0) as a baseline, i.e., $\Delta A(\lambda, t) = \lg(I(\lambda, 0) / I(\lambda, t))$.

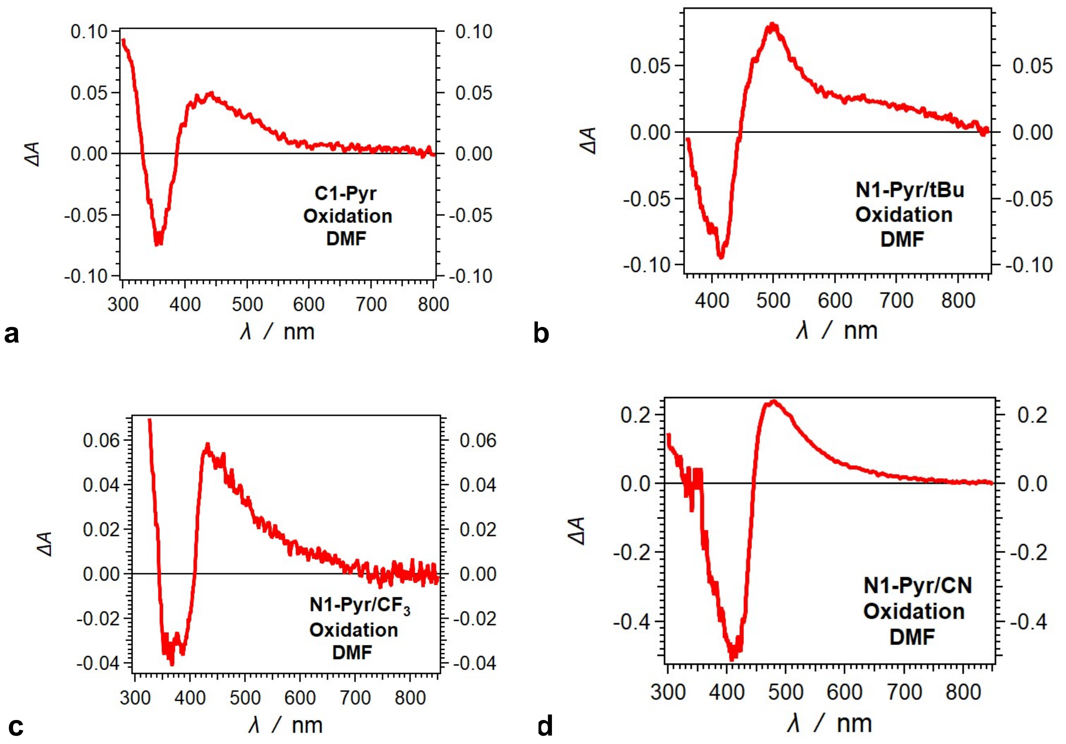

Figure S31. Differential absorption spectra corresponding to radical cations recorded at the first anodic waves during positive cyclic-voltammetry sweeps of (a) ***C*1-Pyr**, (b) ***N*1-Pyr/*t*Bu**, (c) ***N*1-Pyr/CF_3**, and (d) ***N*1-Pyr/CN** in DMF in the presence of 100 mM $N(C_4H_9)_4PF_6$. The close similarity between these spectra of the pyrene-pyrrolopyrrole conjugates and that of TAPP indicate that the radical cation is localized on the pyrrolopyrrole core.

Theoretical analysis: We have performed the DFT and TD-DFT calculations with Gaussian 16.[1] For all systems we kept the actual structures w/o simplification. Default Gaussian16 thresholds and algorithms were used but for an improved optimization threshold (10^{-5} au on average residual forces), a stricter self-consistent field convergence criterion (10^{-10} a.u.) and the use of the *ultrafine* DFT integration grid.

Firstly, the S_0 geometries have been optimized with DFT and the vibrational frequencies have been analytically determined, using the M06-2X *meta*-GGA hybrid exchange-correlation functional.[2] We build the molecules in the C_i point group and this led to stable ground-state minima. These calculations were performed with the 6-31+G(d) atomic basis set and account for solvent effects through the linear-response PCM approach considering toluene and acetonitrile as solvents.[3] Secondly, starting from the optimal ground-state geometries, we have used TD-DFT with the same functional and basis set to optimize the S_1 geometry and compute the vibrational frequencies. These calculations sometimes led to unstable results in the C_i point group and symmetry was lowered. Alternatively, we build molecules with a CT character starting from optimized excited-state and ground-state fragments "plugged" together and checked the relative stabilities of these structures after full excited-state optimization. All optimized structures used in the main text correspond to true minima of the potential energy surface. Thirdly, the vertical transition energies were determined with TD-DFT and the same functional, but a larger basis set, namely 6-311+G(2d,p) using the cLR^2 variant of the PCM,[4] in its *non-equilibrium* limit. From this, we obtained 0-0 energies using a procedure detailed elswehre.[5] The EDD were computed from the relaxed TD-DFT densities, and CT parameters used the well-known Le Bahers approach.[6]

[1] M. J. Frisch, et al. Gaussian 16, revision A.03; Gaussian Inc.: Wallingford, CT, 2016.

[2] Y. Zhao, D. G. Truhlar, *Theor. Chem. Acc.,* 2008, **120**, 215−241.

[3] J. Tomasi, B. Mennucci, R. Cammi, *Chem. Rev.*, 2005, **105**, 2999−3094.

[4] C. A. Guido, A. Chrayteh, G. Sclamani, B. Mennucci, D. Jacquemin, *J. Chem. Theory Comput.,* 2021, **17,** 5155−5164.

[5] D. Jacquemin, A. Planchat, C. Adamo, B. Mennucci, *J. Chem. Theory Comput.,* 2012, **8,** 2359−2372.

[6] T. Le Bahers, C. Adamo, I. Ciofini, *J. Chem. Theory Comput.,* 2011, **7,** 2498−2506.

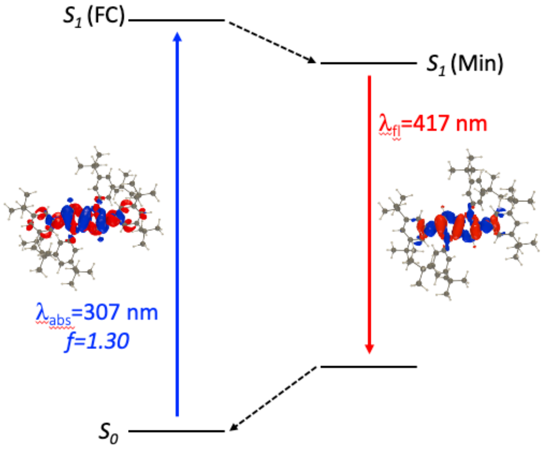

Figure S32. Jablonski-like diagram obtained with PCM(DMF)-TD-DFT for **TAPP**. For both absorption ane emission, we report electron density difference plots (EDDs). Blue and red lobes correspond to decrease and increase of electron density upon transition. Contour: 1 × 10^{-3} a.u.

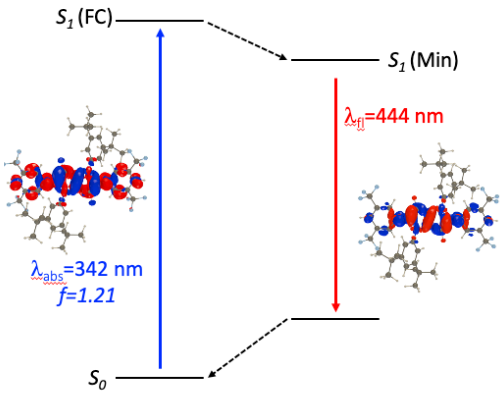

Figure S33. Jablonski-like diagram obtained with PCM(DMF)-TD-DFT for **TAPP-CF_3**. See caption of Fig. S32 for more details.

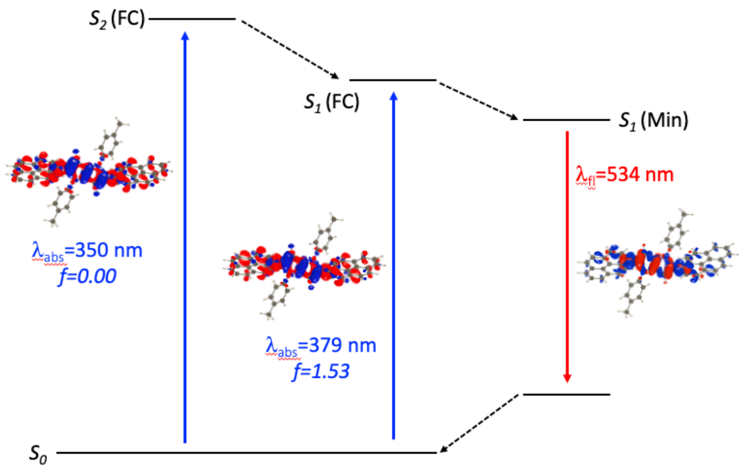

Figure S34. Jablonski-like diagram obtained with PCM(DMF)-TD-DFT for ***C*1-Pyr**. See caption of Fig. S32 for more details.

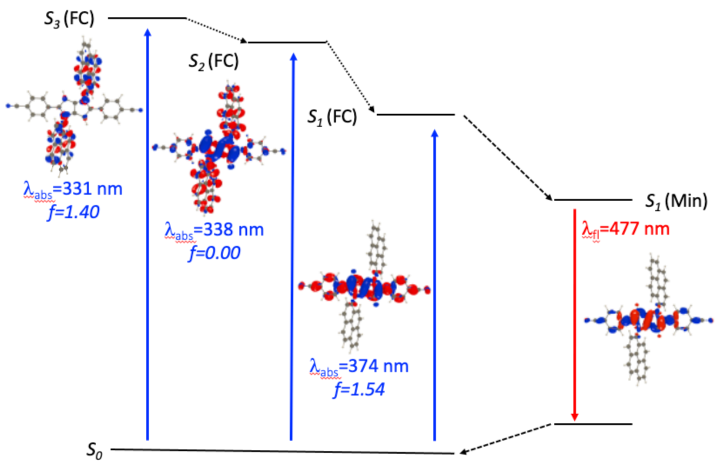

Figure S35. Jablonski-like diagram obtained with PCM(DMF)-TD-DFT for ***N*1-Pyr/CN**. See caption of Fig. S32 for more details.
